# Biomarkers in Japanese Encephalitis: A Review

**DOI:** 10.1155/2013/591290

**Published:** 2013-12-18

**Authors:** Ravi Kant Upadhyay

**Affiliations:** Department of Zoology, D. D. U. Gorakhpur University, Gorakhpur, Uttar Pradesh 273009, India

## Abstract

JE is a flavivirus generated dreadful CNS disease which causes high mortality in various pediatric groups. JE disease is currently diagnosed by measuring the level of viral antigens and virus neutralization IgM antibodies in blood serum and CSF by ELISA. However, it is not possible to measure various disease-identifying molecules, structural and molecular changes occurred in tissues, and cells by using such routine methods. However, few important biomarkers such as cerebrospinal fluid, plasma, neuro-imaging, brain mapping, immunotyping, expression of nonstructural viral proteins, systematic mRNA profiling, DNA and protein microarrays, active caspase-3 activity, reactive oxygen species and reactive nitrogen species, levels of stress-associated signaling molecules, and proinflammatory cytokines could be used to confirm the disease at an earlier stage. These biomarkers may also help to diagnose mutant based environment specific alterations in JEV genotypes causing high pathogenesis and have immense future applications in diagnostics. There is an utmost need for the development of new more authentic, appropriate, and reliable physiological, immunological, biochemical, biophysical, molecular, and therapeutic biomarkers to confirm the disease well in time to start the clinical aid to the patients. Hence, the present review aims to discuss new emerging biomarkers that could facilitate more authentic and fast diagnosis of JE disease and its related disorders in the future.

## 1. Background

Japanese encephalitis virus is a single stranded positive sense RNA virus belonging to family Flaviviridae. It is one of the major causative agents of pediatric encephalitis or viral encephalitis in Southeast Asia. Due to demographic, environmental, and therapeutic reasons, its outbreak commonly occurs almost every year among children [1]. JE is a dreadful zoonotic disease that generates high morbidity and mortality in pediatric groups. Its transmission is seasonal that occurs very fast in rainy season due to mass breeding of rice field mosquito vector, that is, *Culex tritaeniorhynchus summorosus*, especially in undeveloped rural areas. Because of scattered occurrence of JE in different states and regions in India, the actual JE burden and magnitude of infection cannot easily be estimated. Due to lack of proper and timely diagnosis of JEV and extra delay in treatment, very high mortality occurs in various infant groups [2]. However, JE control could be possible only by strengthening diagnostic facilities for its confirmation in hospitals situated in rural areas and by establishing national surveillance system for knowing postvaccination adverse effects. Moreover, an earlier diagnosis of the disease and medical care is required for patients. Nevertheless, patients with mixed and typical symptoms of brain fever or saddleback fever, with low platelet counts and hematocrit values should provide immediate clinical care [3]. This clinical phase is highly important to diagnose the disease. To date a definitive diagnosis of JE can only be made with clinical symptoms, biochemical profiles, and serological examination of JE patients. Therefore, treatment strategies might be most effective before virus pathogenesis spreads throughout brain and spinal cord. Thus, an earlier diagnosis based on reliable biomarkers is essential to identify the status and intensity of JE infection.

The current literature and clinical investigations reveal that climate induced genotypic variations are going on in newly emerging molecular variants of flaviviruses mainly related to encephalitis. It proves that JE virus is making possible modifications in antigenic structure through mutations both in nucleotide and protein sequences. It is very hard to explain environment-induced mutations and other molecular changes occurring in antigenic sites. If identifying these minor differences in mutant strains of JE virus could help to detect environment-specific alterations in pathogenesis in recent past and future. Further, vaccine attenuated sequence alterations are significantly increasing which are not only changing the etiological features of the virus but are also inducing high neurovirulence and host immune responses and affecting disease transmission in both endemic and nonendemic population. However, other than JE virus endemic strains, new vaccine-derived recombinant strains of JE virus have been evolved which are detected in serum samples of patients from endemic regions. However, overall changes occurred in JEV have increased the infection, pathogenesis and mortality rate. It has also led to an increase in expansion of severity of infection in nonendemic areas. Among other possible reasons of expanding its area are global climates, ecological and socioeconomic changes, and mutations acquired by local strains of JE virus.

Though so many methods of JEV detection are available to days, it is very hard to confirm the JE disease based on seeing visible pathological symptoms in patients. However, it is very clear that disease history of JE infection, virus exposure, and certain clinical features cannot help to diagnose this disease properly. There is an utmost need to develop potential biomarkers, which might essentially improve the diagnosis and accelerate the development of new vaccines. More specifically, biochemical, molecular, and immunological tools are considered to be more authentic and reliable to detect the JE virus in different tissues and cells [4]. These markers could facilitate fast diagnosis of JEV invasion in neuronal cells and behavioral impairments in patients and help to confirm progression of disease. However, a clinical diagnosis based on determination of the level of virus IgM antibodies, expression of nonstructural viral proteins, and pathological changes in neuronal progenitor cells, brain, cortex, thalamus, hippocampus and striatum will be more helpful in JE confirmation [5]. Similarly, measurement of the level of certain markers in CSF such as the level of interleukins, complements, cytokines, and intracellular virus proteins was also found to be useful in predicting the JEV generated risks and prevalence of pathogenesis [6]. However, definitive diagnosis of JE based on clinical symptoms, serology, and biochemical profile of the virus is quite investigative [7], but it is very difficult to diagnose on going mutations in newly emerging genotypes of JE virus circulating in the endemic population. Further, genotype-based neurovirulence, antigenicity, pathogenesis and mortality must reinvestigated by using new candidate markers. Biomarkers will assist in exploration of pathological mechanisms followed by virus during invasion, persistence and clearance. Therefore, strong molecular and protein biomarkers are needed for the confirmation of genotype-based infection of JE virus in patients as well as its therapeutic essentiality accordingly.

However, biomarkers based on measurement of a highly specific biomolecule that may confirm the presence of JE virus pathogen inside host will clearly display the physiological state of patient. Biomolecules, synthesized in response to JE virus attack and cellular invasion will show clear biological characteristics whose measurement in body fluid, cells and tissues will confirm the viral disease [4, 5]. For example, formation of C-reactive protein (CRP) in response to JE virus attack works as a marker for inflammation. Further, all affected specific cells, molecules, or genes, gene products, enzymes, or hormones, complex organ functions, characteristic changes that occurred in the biological structure, and function of Biomolecules could serve as biomarkers. Therefore, biomarkers that clearly indicate and reflect the severity and status of viral infection in patents will be more appropriate. More specifically, such biomarkers could correlate virus-induced changes with the risk and progression of JE disease and susceptibility of virus to a given treatment. Similarly, imaging biomarkers (CT, PET, and MRI) and nonimaging biomarkers could also explore biophysical properties of virus and bio-molecules with their measurements in biological samples (e.g., plasma, serum, cerebrospinal fluid, brain biopsy).

More specifically, nucleic acid-based biomarkers such as gene mutations or polymorphisms and quantitative gene expression analysis, virus-induced peptides, proteins, lipids metabolites, and other small molecules could help in early diagnosis of JE. However, gene-based biomarker will be more effective and an acceptable marker and may prove better JE diagnostic tools for fast clinical assessment of JEV in the future. Furthermore, subset of markers that might discover secretes of virus invasion and cellular pathogenesis by using genomics, proteomics technologies or imaging technologies would play major roles in medicinal biology. Further, strong therapeutic pharmacodynamic (PD) biomarkers will be required for decision making and drug development, pharmacological response, and dose optimization. However, to have an early clinical investigation, ready highly specific biomarkers are essentially required to display the JE infection and physiological state of patient before starting certain medicare. Hence, in the present review article newly emerging biomarkers for JE are highlighted with their diagnostic efficacy specifications, authentication and working accuracy with an objective to recognize cellular abnormalities occurring in the JE infected patients. These biomarkers might prove to be significant diagnostic tools for JE confirmation in the future.

## 2. Cerebrospinal Fluid Biomarkers

JE virus cultured in the host body cells releases so many components in the cerebrospinal fluid, which serve as biomarkers. Hence, both interacting and noninteracting molecules released by the cells in anticipation to virus load and invasion of blood and nerve cells are determined accurately. However, alot of virus eradicating molecules are released by immune cells and virus toxins/antigens in the CSF are assayed for evaluation of presence of virus, intensity and rate of infection in human hosts. However, for detection of JE virus interacting molecules in cerebrospinal fluid and blood serum samples are collected from both JE endemic and epidemic areas. In normal population, blood serum samples are collected in the premonsoon season and at the time of outbreak. During infection, season samples are collected twice at a time interval of ten days. Patient information should be recorded with 5–10 different specifications; it should include location, name, age, sex, vaccinated or not, date of onset of symptoms, types of specimen, date of collection, economic group, viability, and medicare given. Experienced personnel under aseptic conditions should do sample collection. CSF collection should be done in separate vials for each bioassay related to biochemistry, microbiology and virology of the JEV. Normally for each investigation 0.5–1 mL of CSF is required. The sample must be kept at 4°C for short-term storage, but for long-term storage this sample must be kept below −20°C. Repetitive freezing and thawing should be avoided. Liquid nitrogen containers are used for transportation of samples to the investigating laboratories.

However, level of virus neutralization antibodies in CSF works as one of the important biomarkers for knowing the presence of JE virus or any other viral infection. For measurement of neutralizing antibodies level, in cerebrospinal fluid (CSF) and serum, few conventional techniques such as viral neutralization, hemagglutination inhibition (HI), complement fixation tests and immune-florescent staining methods are mostly applied. All these techniques showed limitations as routine diagnostic tests, because these are labor-intensive, expensive, cumbersome, and not sensitive to the detected antibodies in CSF [5]. These virus neutralization antibodies successfully block viral infection by neutralization, after formation of virus- antibody complex. This complex can prevent viral infection in many ways. But, success rate in clinical specimens remains less because of the low level of viremia and rapid development of neutralizing antibodies (Table 1) [8]. Recently, much potent neutralizing human antibodies have been synthesized against JE virus to measure the level of circulating virus in CSF and other body fluids of patients. These neutralizing human antibodies help in more accurate sero-diagnosis of JE virus in clinical samples [5]. In addition to this, rapid micro-neutralization test (MNT) is also developed to detect neutralizing antibodies to JEV virus in the CSF of the Japanese encephalitis patients [4]. It also helps to establish single virus infection in patients [9]. It is performed by using maximum dilution of antibody which can confirm 90% reduction in viral infectivity after virus neutralization [10]. Similarly, reduction neutralization test (PRNT) helps to detect humoral immune response generated after immunization with JE inactivated vaccine [6]. Thus, both MNT and PRNT detect Japanese B encephalitis virus (JEV) neutralizing antibody titers more efficiently and these titers work as strong markers of JE disease [11]. These tests also confirm low JE infection level by measuring neutralization antibody titer [12]. However, early and high neutralizing antibody responses are crucial for preventing viral neuroinvasion and host fatality [13]. At this stage, virus generated Biomolecules if assayed may work as strong and useful marker to diagnose the JE disease at an earlier stage. However, virus neutralization antibodies, mainly polyclonal antibodies, can subvert the attack of JE virus [7].

Further, the presence of viral encephalitis is also detected by analyzing titers of neutralizing antibodies by DEIA and other techniques such as CT, MRI, EEG, spinal tap and brain biopsy (Table 1). Further, neutralizing antibodies synthesized against nonstructural neurovirulent proteins may be more helpful in disease diagnosis. These antibodies remain detectable in CSF and blood within 7 days after onset of disease [14] by IgM capturing ELISA more accurately [15] and authentically (Table 1) [16, 17]. Few other methods like immunological haemagglutination inhibition [18] and complement fixation tests accurately detect presence of JE infection. Further, immunotyping techniques based on antibody absorption precisely determine minor variations in various immunotypes prepared against different virus antigens [19]. For example, by using this method 2 strains of Nakayama-NIH and Nakayama-Yakken immunotypes were identified. The Nakayama-RFVL strain was found to have the characteristics of both immunotypes while I-58 immunotype differs more markedly from related arboviruses, such as the Murray Valley encephalitis virus and the West Nile Eg101 strain. Moreover, the HI test is widely used for the diagnosis of Japanese encephalitis virus, but shows great limitation and fail to detect cross-reactivity with other flaviviruses. More specifically, sera treated with acetone or kaolin, and then adsorbed with homotypic RBCs are used to remove any nonspecific haemagglutinins [18] (Table 1).

However, glycoprotein E (V3) of different viruses, which expresses antigenic determinants and its differential binding to antibodies, is used in haemagglutination (HA) assay. Further, display of antigenic relationships of the E protein among several flaviviruses, that is, WN subgroup viruses (JE, MVE, WN, and SLE) and other subgroup flaviviruses (DEN) MAbs prepared against the E protein of JE virus is used [19]. More specifically, epitopes of E protein of several flaviviruses such as JE virus [20], tick-borne encephalitis (TBE) virus [21, 22], SLE virus [23, 24], YF virus [25], WN virus [26], and DEN virus [27] are used to synthesize different virus-specific Mabs. These antibodies can explore cross-reactive sites in genotypes and authenticate presence of JEV among all the flaviviruses more strongly. These will also help in clinical establishment of the cause of JE disease in particular areas. Moreover, serial measurement of serum NS and E proteins might be a useful marker for diagnosing the disease and therapy. A plasmid DNA vaccine encoding prM-E protein from the JE virus also elicits cellular immune responses. Further, significant homology in amino-terminal amino acid sequences of E proteins of different flaviviruses by using amino acid sequencing and proteome analysis can also establish status of antigenicity, neurovirulence, and disease pathogenesis [28].

However, ELISA is a more accurate method, which can detect nanogram quantity of JE specific antibodies in patient's blood serum and replaces both serum neutralization and HI tests because of its high sensitivity and binding to virus antigens [8]. Moreover, fast diagnostic automation JE ELISA test is also developed and authenticated to use antigen JERA. JERA is a recombinant antigen that consists of a stretch of peptides from different parts of JEV antigens [29]. It is used as a rapid serological marker for detection of JEV-specific antibodies in patient's blood. Similarly, another JEV IgG ELISA is also developed. It is a two-step sandwich-type immunoassay (Table 1) relatively more rapid and reliable method that can accurately diagnoses JE virus with a single specimen collected during acute phase [30]. It is used in field evaluation of circulating JE virus among encephalitis patients [31] and is routinely used as serological in-house assay to measure JEV specific IgM antibodies. However, flavivirus group shows intense cross-reactivity to IgG level but secreted IgM level in CSF can detect JE virus more accurately [30].

Similarly, a dipstick enzyme-linked immunosorbant assay is also used for detection of JE virus-specific IgM antibodies [8] (Table 1). It shows very high sensitivity and specificity to JEV and is used as a promising diagnostic tool in field conditions. It is routinely used for laboratory diagnosis of JE virus. It is a simple rapid test and requires no specialized equipment. Similar to dipstick ELISA, MAC-ELISA is also used as a valuable diagnostic tool that detects secondary flavivirus infection in comparison to hemagglutination inhibition test [8]. It was found to be very sensitive and highly specific with more than 90% confidence. MAC assays have one great advantage over conventional indirect assays based on IgG antibodies. IgM detection shows higher sensitivity in MAC ELISA, which shows ratio of 1 : 300 in diseased and in apparent infection [32]. MAC ELISA clearly provides difference among JEV and DEN virus IgM antibodies and diagnosis can be made from a single sample (preferably CSF) collected during early acute phase of infection [30]. Similarly, NIMHANS Bangalore, NII New Delhi, and K. G. Medical College Lucknow developed JEV diagnosing kit, JEV-Chex under DBT umbrella program. Chex is a rapid ELISA kit for the detection of IgM antibodies in human CSF and serum. Similarly, few commercial JE detection kits such as Euroimmun anti-JEV IgM IIFT, and the Panbio Japanese Encephalitis—Dengue IgM Combo ELISA are also available in the market which can detect JE virus infection more accurately. These bioassays show more than 90% specificity and sensitivity. These are highly reliable established methods to detect JEV infection in travelers and common people (Table 1). However, IgM antibody level is one of the important markers, which can more precisely investigate JE virus in acute phase of infection in CSF of patients [30]. More usually, measurement of the rates of infection can be determined by observing seropositivity in mosquitoes and birds.

## 3. Serum Biomarkers

Serum protein profiles work as potential biomarkers for knowing infectious disease status in animals [33]. These are generated by SELDI-TOF mass spectrometry in combination with the ProteoMiner technology that accurately displays low-abundance proteins responsible for virus infection. These also clearly display status infectious disease and rate of infection in separate models or hosts. Similarly, potential biomarkers for a number of human and animal diseases are facilitated by proteomic analysis of serum proteins and enzymes [10]. However, comparative proteomic analysis of serum proteins on SELDI-TOF-MS [11] and chip arrays could find differences in virus and host secreted proteins during various interaction periods. Such arrays could bind intact proteins present in biological samples, such as body fluids or tissue extracts and detect virus-induced effects. Such arrays may vary in their surface chemistry, for instance they may have hydrophobic or hydrophilic properties, and thereby selectively bind proteins that could be identified by their specific molecular weights. However, correlation between SELDI-TOF MS results and clinical data could recognize significant variation in virus specific proteins components that differ in abundance between groups of samples. Therefore, quantitative data of high- and low-abundant serum protein components measured by SELDI-TOF-MS can be used for early detection and diagnosis of viral infectious diseases. These protein profiles could alternatively obtained in other biological samples like saliva, urine, or feces. Further, recent developments that occurred in the field of micro- and nanotechnology created larger interest for researchers to develop sophisticated electronic devices for clinical health monitoring. However, several promising prototypes are emerging in human biomedicines mainly for diagnosis of patients with neurological diseases [23].

A quantitative microcomplement fixation test also detects nanogram quantities of antigen in serum blood samples of JE virus infected patient. The test is used to detect the presence JE virus specific antibodies in serum and is highly reproducible. Normally after seven days of transmission of JEV virus, B cells produce antibodies during an active infection, which defend the body against viruses and other foreign substances, called antigens. If the antibodies are present, they attach to the antigen. This combination activates or “fixes” complement. The test is more useful to know the rate of infection by determining the level of serum antibodies with the help of specific antigen. Complement binds to antigen-antibody complex and leads to cell lysis. Complement subsequently binds to this antigen-antibody complex formed and will cause the red blood cells to lyse [34]. If the patient's serum does contain a complement-fixing antibody, a positive result will be indicated by the lack of red blood cell lysis. Besides this, measurement of glucose, protein level and mononuclear white blood cell counts done in CSF samples which were obtained by lumbar puncture also confirm presence of virus. The CSF rarely yields virus, except in severe or fatal cases, but in full blooming JE infection, serum antibody level raises up to 4-fold, whose measurement is only possible by CSF analysis. Besides this, a complete blood count (CBC) often helps to detect leukocytosis, leucopenia, anemia and thrombocytopenia in JE patients [35] (Table 1). However, sequential changes in serum cytokines chemokines work as good biomarkers for JE virus [36]. There were increased levels of proinflammatory and anti-inflammatory cytokines and a chemokine (monocyte chemoattractant protein-1) in the serum of rats after JEV infection compared to controls [36]. However, significant alteration levels of cytokines and chemokine peaked at 10 dpi and declined significantly by 20 dpi which shows neurological invasion in the acute stage of disease and partial recovery thereafter [36].

## 4. Plasma Biomarkers

Cells infected by viruses express viral antigens on their membranes long before the viral assembly takes place. If a CTL and a suitable antibody are being made available with supporting active complement proteins, it destroys large population of virus. It is a very effective mechanism, which helps in mass destruction of virus-infected cells by using antibodies (antibody-dependent cell mediated cyto-toxicity ADCC system), classical pathway of complement activation, phagocytosis, and cytotoxicity mediated by CD8+ lymphocytes. Thus, formation of antigen-antibody complex stimulates the Fc receptor on macrophages (CTLs) that helps in viral clearance and evokes heavy complement mediated cell lysis of virus or virus infected cells [37]. However, in response to a virus attack, complement induces synthesis of proinflammatory peptides (3a and 5ca) which help to reunite and activate monocytes and granulocytes to the inflammatory site. Further, proteolytic fragments C3 (C3b, C3bi, C3d and C3dg) promote uptake by cells that express complement receptor [38]. In this process, C3 convertase enzyme helps and catalyzes the reaction. Thus after amplification hundreds of C3b molecules are generated which bind to nearby cells and mediate damage to healthy cells by opsonization to phagocytic cells [39]. C3b receptors help to form membrane attack complex. There is another possibility that C3 fragment enhance viral antigen uptake, facilitate antigen presentation by macrophages and DC, and induces specific antibody production and T cells proliferation [40]. More specially, C3b, C4b, and C3bi fragments play important role in opsonization of antigen while C3b and C5b-9 help in neutralization of virus with C3b, C5a, and C5b67 causing extravasations and chemotaxis of neutrophils and monocytes [41].

In addition, a number of cytokines play a significant role in the development of an acute or chronic inflammatory response, and IL-1, IL-6, TNF-*α*, IL-12, and many chemokines exhibit redundant and pleiotropic effects that work together and contribute to the inflammatory response [42]. The inflammatory response provides early protection following infection or tissue injury by restricting the tissue damage to the affected site. The acute inflammatory response involves both localized and systemic responses. Similar to JE Dengue virus flavivirus also show pathogenesis by chemokines and cause severity of infection as virus associates to chemokine receptors CCR1, CCR2, and CCR4 [43]. Dengue virus infection also induces clinical symptoms related to tissue damage like thrombocytopenia, hemoconcentration, lymphopenia, increased levels of transaminases and proinflammatory cytokines. It also shows antibody-dependent enhancement of growth in human monocytes that is a serious risk factor in hemorrhagic fever [44]. Besides this, adhesion mechanisms regulate the migration of monocytes [45] and disease severity increases with systemic inflammation and activation of chemokine receptors, which play discrete roles in the pathogenesis. Besides chemokines, a variety of other mediators released by cells of the innate and acquired immunity also trigger the inflammation. Other disease-causing viruses like JE virus may follow the same process in primary infection and in generation of pathogenesis. Similarly, in vaccinated population, levels of IL-6, IL-8, MCP(1) (monocyte chemo.-attractant protein), MIP-1a, and MIP 1b (macrophage inflammatory protein) were found to be significantly higher which also play important role in the cellular immune responses to JE. There is another possibility that JE virus inhibits the formation of human monocyte-derived macrophages to chalk out their phagocytic function [46]. Thus, a virus-generated biomolecule during primary infection may become a strong biomarker by recognizing its level in control and early-infected patient [47].

Plasma contains immunoglobulins, enzymes, lysozyme, and properdin a large protein. All these plasma proteins serve to destroy microorganisms including viruses and toxic substances that may enter into the blood from outside or from body tissues. However, mast cells continuously release heparin a conjugated polysaccharide that serves to prevent coagulation of blood during its circulation. Albumins occur in plasma are mainly responsible for osmo-regulation in cells and tissue fluids. Similarly, various ions viz. chlorides, carbonates, phosphates, sulphates and iodides of calcium, magnesium and potassium maintain electrolytic functions. Plasma also contains four interconnected mediator-producing systems which act through activation of G-protein-coupled constitutive or inducible receptors linked to signaling pathways involving increased intracellular Ca(++) concentrations and/or release of mediators including arachidonic acid metabolites. These important systems are kinin system, the clotting system, the fibrinolytic system, and the complement system. More specifically, Hageman factor (factor XII) performs an intermediate function in the first three systems commonly when excessive damage occurs in the tissues. These four systems activate to form a web of interacting processes that generate a number of mediators in inflammation and have great pharmacological role. Both, plasma and tissue kallikrein-kinin system work together and maintain pharmacological properties while kinin receptors and drugs reported to interfere with their actions. By maintaining unique inter-relationship, these mediators induce smooth muscle contraction and increase vascular permeability C3a, C5a, and C5b67 and act together to induce monocytes and neutrophils to adhere to vascular endothelial cells to maintain extravasasive activity through endothelial lining of the capillary and migrate toward the site of complement activation in the tissues [48]. Activation of complement system results in influxes of fluid that carry antibody and phagocytic cells to the site of antigen entry [49]. Some lipids, mainly phospholipids, also act as inflammatory mediators, which include thromboxanes, prostaglandins, leukotrienes, and platelet activating factors [42]. Following membrane perturbations phospholipids are degraded into arachidonic acid and lysoplatelet activating factor when subsequently converted into platelet activating factor that causes platelet activation and induce inflammatory effects, changing eosinophil chemotaxis and the activation of granulation of neutrophils and eosinophils. Acute phase proteins and other systemic responses also play an important role in inflammation [50]. However, its elevated concentration if measured by ELISA in serum could be used as a strong biomarker.

In addition, plasma also contains prothrombin and fibrinogen, which help in blood clotting. Moreover, a kinin system is an enzymatic cascade that begins when a plasma clotting factor called Hageman factor (factor XII) is activated following tissue injury. This activated Hageman factor activates prekallikrein to form kallikrein, which cleaves kininogen to produce bradykinin which increases vascular permeability, causes vasodilatation, induces pain, and triggers contraction of smooth muscles. Kallikrein also acts directly on the complement system by cleaving C5 into C5a and C5b. Another enzyme cascade that is triggered by damage to blood vessels yields less quantity of thrombin [51]. However, initiation of inflammation response also triggers less clotting system and yields fibringenerated mediators of inflammation. More specifically, the fibrino-peptides act as inflammatory mediators, and induce increased vascular permeability and neutrophil chemotaxis. Activated platelets release CD4OL, which leads to increased production of proinflammatory cytokines IL-6 and IL-8 and increased expression of adhesion molecules. The integrin CD11b/CD18 (MAC-1) also plays important role in clotting and binds two components of the clotting system, factor X and fibrinogen. Binding of factor X and CD11b/CD18 increases the activity of factor X, thereby promoting coagulation. The fibrinolytic system yields plasmin-generated mediators of inflammation that initiates the inflammation response by activating the classical component pathway. Anaphylotoxins thus produced from complement system bind to mast cell membrane receptors and induce de-granulation after release of histamine and other pharmacologically active mediators. These factors also act as opsonins and chemotactic molecules for neutrophils and monocytes. However, several types of mediators play a role in the inflammatory response, in which chemokines act as chemoattractants and activate molecules during extravasations.

Nevertheless, antibody and complement that play a role in host defense against viruses are often crucial in containing viral spread during acute infection. However, more or less, all enveloped viruses are susceptible to complement mediated lysis. The viral envelope is largely derived from the plasma membrane of infected host cells and is therefore susceptible to pore formation for the membrane attack complex. The membrane attack complex formed by complement activation can lyses viruses and infected cells. In addition, JEV antigens associate with complement binding receptors and cell adhesion molecules present on the surface of neutrophils accurately detect cellular invasion of virus on neuronal cells, de-granulation and B cell phagocytosis [52]. Further, cleavage products of complement components C3a and C5a are called anaphylatoxins which can be used as biomarkers, if they bind to receptors on mast cells and blood basophils and degranulation.

Furthermore, complement system also mediates neutralization of viral infection by forming larger viral aggregates. It is also supported by antibodies when forming a thick coat around virus particle that neutralizes viral infectivity by blocking attachment to susceptible host cells. If the deposits of antibody and complement on virus particles are detected can give accurate results and become an important biomarker in JE disease. It also facilitates binding of the virus particles to cells possessing Fc or type 1′ complement receptors. Viruses have developed a number of different strategies for evasion of membrane complex attack and natural immunity [53]. These foil the potential MAC activity by making interference with the binding of complement to antibody-antigen complexes, mimicking mammalian complement receptors in the virion. However, interactions of complement proteins to the virus and host immune cells can sketch status of localization of virus generated tissue damage in SEV and other regions with electron micrography, which potentially help to detect pathological signs. Further, presence of JEV cell adhesion molecules (CAMs) and virus specific binding can also work as JEV marker.

However, radio labeling techniques allow sensitive detection of antigens or antibodies and other inflammatory molecules (Table 1). Moreover, inflammatory cytokines released in response to infection can be measured by radio labeling methods both inside body fluids and inside the host body cells. However, determining the levels of inflammatory cytokines, mainly TNF-*α*, can express upregulation of inflammatory cytokines [54]. Moreover, number of CTLs formed and secretion of IFN *γ* from CD8 cells and binding its to NK cells induces lytic activity [55]. Similarly, biotin labels facilitate detection of small amounts of proteins by ELISA or ELISOT. Two-photon microscopy is also found capable of optically sectioning the material under examination without causing phototoxic damage. This technique allows the tracking of cells in their biological environment overtime providing a temporal view of the behavior of lymphocytes following manipulation of the immune system. Furthermore, green florescent proteins and their derivatives are used to analyze presence of living cells and dead cells in tissues. Similarly, by using CFSI2 fluorochrome 5,6-carboxy fluoresciin diacetate succenyl ester techniques labeling of important viral proteins in tissue and cells become possible. Similarly, labeling of antibodies with biotin and avidin allows accurate determination of the level of antibody response during disease and nondisease state [56]. All these are emerging biomarkers, which help to establish the virus-generated effects in human and animal hosts.

## 5. Imaging Biomarkers

Neuroimaging constitutes an important component in the diagnosis of the underlying infectious agents in the central nervous system infection. Many new biomarkers are developed that involve imaging technology to display cellular and tissue injuries in the central nervous system diseases. Imaging biomarkers have many advantages, as they focus on imaging of viral encephalitis, including that caused by exotic and emerging viruses. Imaging biomarkers are usually noninvasive and generate intuitive, multidimensional results on both qualitative and quantitative data. If combined with other sources of information, imaging biomarkers can provide more accurate structural effects of viruses in infected patients to clinicians and diagnose more authentically encephalitis syndromes. These are noninvasive and relatively comfortable for patients.

Magnetic resonance imaging (MRI) and computed tomography (CT) are noninvasive neuroimaging techniques which are used for detection of bilateral thalamic lesions with hemorrhagic regions and other structural abnormalities in basal ganglia, putamen, spinal cord, and cerebellum (Table 1). However, to identify JEV generated hyperintense lesions in thalamus, cerebrum, and cerebellum T2-weighted MRIs are used [57]. In addition, electroencephalography (EEG) also reveals diffuse delta pattern with spikes, theta waves, and burst suppression in nerve cells (Table 1). These methods could help to establish JE virus selective infection in the neurons, causing of ultrastructural changes in association with viral replication in the cellular secretory system, principally involving rough endoplasmic reticulum (RER) and Golgi apparatus [58]. In the early phase of infection, RER of infected neurons showed hypertrophic changes, containing assembling virions within its dilated cisternae. In the later stage, the SER became cystic and degenerative due to transport of multiple virions from Golgi apparatus to RER cisternae, which was later on released into the cytoplasm with in coated vesicles for exocytosis [58]. JE virus infection initiates endoplasmic reticulum stress and an unfolded protein response [59]. In the late phase of infection, host body shows some regenerative changes in membranous organelles [58].

Similarly, diffusion-weighted imaging or diffusion tensor imaging is proved to be superior to conventional magnetic resonance imaging for the detection of early signal abnormalities in herpes simplex virus encephalitis but also in enterovirus 71 encephalitis and in West Nile encephalitis. More specifically, diffusion signals capture micro-structural properties of brain white matter, but it is not feasible by structural MRI scans. However, pattern of diffusion-weighed imaging signal changes in endemic diseases such as West Nile encephalitis, Murray Valley encephalitis, enterovirus and Japanese encephalitis is a newly emerging biomarker. However, apparent diffusion coefficient ratios obtained by diffusion-weighted imaging confirm patients with HIV infection [60]. Similarly, surface-enhanced Raman scattering SERS can deliver chemical and structural information from analytes rapidly and nondestructively in a label-free manner. Alternatively, SERS labels or nanotags, when conjugated to target-specific ligands, can be employed for the selective detection and localization of the corresponding target molecule. It may have wider application in neuroimaging of CNS disease viruses like JE [61].

Other imaging techniques used are magnetic resonance imaging (MRI), optical coherence tomography (OCT), near infrared spectroscopy, radio labeled fludeoxyglucose, position emission tomography (PET), and diffusion tensor imaging which more exceptionally detect any type of atrophy that occurred in temporal lobes, cerebral cortex, thalamus, and brain stem. In addition, structural changes neurophysiological impairments are also determined by measuring take-up glucose in the body cells. However, tracking glucose, sites of inflammation can easily be explored because macrophages maintain high levels of take-up glucose. However, high utilization of glucose in the state of tumor growth or during cellular necrosis that occurred due to invasion of viral toxins could be explored by using imaging strategy. MRI provides high spatial resolution and is very adept at morphological imaging and functional imaging. MRI shows a sensitivity range from 10^−3^ mol/L to 10^−5^ mol/L that is very limiting. However, for achieving molecular imaging of disease biomarkers using MRI, targeted MRI contrast agents with high specificity and high relaxivity (sensitivity) are required. For this, purpose, commonly, peptides, antibodies, or small ligands, and small protein domains, such as *HER-2* affibodies, have been applied to achieve targeted imaging. Functional imaging help in measurement of acetylcholinesterase (AchE) and butylcholinestrase activities, nicotinic, muscarinic receptor binding, vesicular acetylcholine transporter, and behaviors and action of neuromodulators in undismayed and diseased individuals. These enzyme-based biomarkers are suggested to be more sensitive and may help to decide early structural changes in brain of JE infected patients [62].

## 6. Anatomical Markers

JE is a severe neurological disease, which causes high fatality in infant groups. Virus generates neurotrophic effects that result in encephalitis syndrome or acute susceptibility to CNS [63]. JE virus mainly targets cerebellar Purkinje cells and causes neurological signs such as ataxia [63, 64]. It also target NCPs pools [65, 66] inhibit cell growth, proliferation [67] and cycle progression [68]. It starts by invasion and destruction of immune cells by cytolytic mechanism which mostly target NCPs pools [69]. It results in a large number of neuronal cell deaths, which occur due to microglial activation and robust inflammatory attack made by JEV virus [70, 71]. However, without knowing structural modifications in neuronal, vascular, and muscular deformities due to JEV invasion, it is very difficult to find more appropriate, authentic and suggestive outcomes for therapeutics and of JE vaccination. Besides this, serological findings are sometimes confusing because of high degree of cross-reactivity amongst the flavivirus antigens. More exceptionally, anatomical markers of JEV generated changes in thalamus, substantia nigra, brain stem, hippocampus, cerebral cortex, cerebrum and spinal cord [72]. With the help of biomarkers invasion of nerve cells, virus transfer from blood stream to brain and occurrence of transient viremia could also be identified. However, immunohistochemical methods clearly display neurophysiological changes in Japanese encephalitis patients [73].

JEV is a neurotropic virus that also targets developing CNS and infects embryonic NPCs and replicates, inhibiting their growth and cell differentiation [67]. JEV infection leads to massive neuronal cell death [74] (Misrha and Basu) and causes severe neuropathogenesis [75]. JEV infected cells show extended lag phase during growth [76, 77]. JEV decreases the number of colony forming NPCs (NPCs pools) and reduce their self-renewal capacity and proliferative ability [68, 69]. A high necrotic cell death was observed in JEV infected NPCs in comparison to control [69]. JE virus also causes morphological changes in NPCs cells in infected patients and animal models, which are localized by using nestin as a marker in double immunohistochemistry [69]. However, nestin positive cells in the JEV infected brain localize two kinds of cells, that is, oval shaped cells and round cells. Round cells are specific markers of virus infestation. Moreover, FACS analysis of BrdU incorporated cells show a significant decrement in its counts in cells from JEV infected subventricular zone of brain [69].

However, as neuronal stem cells are self-renewable [78] but JEV infection shows aberrant formation of neurospheres in NPCs with progressive infection [69]. Just after cellular invasion primary NPCs form secondary neurospheres which are identified by clonogenic assay [79]. Neurosphere are free-floating eight-to-ten cell aggregates and their size become smaller in case of JEV infection. However, a more significant decrease in the number of spheres generated from NPCs of JEV infected subventricular zone than controls *in vivo* assay [69]. In addition, the apoptotic population of nerve cells is detected by YUNEL assay. Both flow cytometry of Annexin V propidium iodide stained NPCs and TUNEL assay revealed high number of apoptic cells with disrupted membrane to morphological alterations and necrosis are other confirmatory points for JEV invasion. BrdU incorporation *in vitro* assay proved that JEV infection inhibits DNA synthesis and cell cycle progression in NPCs. Further cell cycle kinetics in NPCs is analyzed by labeling with 7-ADD [80], a florescent DNA binding dye. Among JEV infected cells most of the cells are found in G0/G1 phase of cell cycle [81] which is an important cellular marker. It indicates that JEV infection blocks cell cycle progression through S. phase in NPCs. It also leads to up-regulation of G>S phase checkpoint proteins [69].

Interaction of dendritic cells (DCs) with innate lymphocytes (NK and NKT) represents a crucial event during antiviral innate immune response. Similarly, IL2-activated CD56(+) lymphocytes mediated immunomodulation and TNF*α*, generate anti-viral effects during direct cell-to-cell contact [82]. However, modulation of cross-presentation of exogenous antigens through TLR signaling plays important role in anti-viral immune responses against JEV infection and may help in development of effective vaccination strategy in the future [83, 84]. Similarly, ICAM-1 (intercellular adhesion molecules) enhanced T-cell receptor signaling and activated Th1 immune responses in the JEV model system by increasing the induction of CD4(+) Th1 cell subset and activating dendritic cells [85]. Further, gene expression is affected by TNF*α* and IL-1*β* produced by JEV-infected microglia during the course of infection. Before invasion virus attaches to host microglial cells with a high affinity to laminin receptor protein and nucleolin which are potential JEV binding proteins [86]. By assaying the antibody inhibition of infection, both antilaminin receptor and anti-CD4 antibodies significantly reduced virus entry [87]. It acts as a strong biomarker that indicates involvement of multiple receptor protein that mediate the entry of JEV to microglial cells with CD4 having a major role in it.

JEV is a neurotropic remerging virus that mainly targets neurons, enters microglial cells, a neuronal cell type [86] and causes massive neuronal destruction/dysfunction [87]. Virus attacks neuronal cells, causing high inflammation in the CNS and try to impair functional and structural integrity of BBB and other regions of brain. BBB (blood-brain barrier) preempts the damage to CNS from exogenous molecules mainly virus generated toxins. Similarly, astrocytes play key role in regulation of inflammation and homeostatic maintenance of the central nervous system [88] (Mishra et al., 2007). JEV infection increases the expression of astrocyte specific glial fibrillary acidic protein (GFAP). Successively after JEV infection both nerve growth factor (NGF) and cellular neutrophin factor (CNTF) are also elevated which prevent ROS mediated neuronal cell death in JE infected host [88]. If the protective efficacy of astrocytes to JE is amplified, it will modulate the adaptive response of the brain to induce higher neuroprotection [88].

Severe dengue virus (DENV) disease is associated with extensive immune activation, characterized by a cytokine storm. However, previously elevated levels of lipopolysaccharide (LPS) in dengue virus patients are found to be correlate with clinical disease severity [89]. Similarly, JE virus caused severe neuroinflammation, which start with robust expression of proinflammatory cytokines and chemokines with increased number of infiltrating inflammatory cells into the brain. The virus mainly infects neuronal cells and causes an inflammatory response after invasion of the parenchyma of the brain. Histopathology confirms the infiltration of leucocytes and there was a marked upregulation in the expression of genes relevant to infiltration. It is also associated with involvement of monocyte and macrophage receptor CLEC5A in severe inflammatory response in JEV infection of the brain. However, expression level and molecules of neuroinflammation can work as important biomarkers for development of appropriate future diagnostic tools for JE therapeutics, and prophylactics [90]. The death of neurons is frequently observed, in demyelinated axons, which is ambiguous. However, presence of myelin-specific antibodies in sera in mice with evident symptoms shows presence of virus in neuronal cells. Further, it is strengthen by specific T cells proliferating in response to stimulation by myelin basic protein (MBP) in mice. It shows autoimmunity that may play an important role in the destruction of components, for example, MBP, of axon-surrounding myelin, resulting in demyelination in the mouse brain after infection with the JE virus [91].

Minocycline, a semisynthetic tetracycline, has been found to be broadly protective in neurological disease. It mainly shows neuroprotective role and slow down inflammation and cell death in experimental models [92]. However, a breakdown in BBB is detected by finding leakage of protein-bound Evan's blue dye into the brain tissue of experimental animal. Semiquantitative RT-PCR revealed an upregulation of chemokine receptors and adhesion molecules following JEV infection. Immunostaining showed leukocyte and neutrophil infiltration following JEV infection. Intraperitoneal injection of minocycline, beginning 24 h after-JEV infection, abrogated the effects by reducing BBB damage, decreasing expression of iNOS, Cox-2, and VEGF, and also by reducing the elevated level of transcript of chemokine receptors and adhesion molecules in the brain. Matrix metalloproteinases (MMPs) are known to disrupt the BBB and minocycline was found to significantly decrease the activity of MMP-9 in brain tissue homogenates and appears to maintain blood-brain barrier integrity following JEV infection [92].

JE virus infection evokes acute encephalopathy in children a clinical syndrome with high mortality and neurological sequelae [47]. Virus invasion in patients generate symptoms such as impaired consciousness and convulsive status epilepticus with hyperpyrexia [93]. It is characterized by detection of biphasic seizures and late reduced diffusion (ASED) in MRI tests [93]. However, there is no specific biomarker for early diagnosis of acute encephalopathy syndrome available, but tau protein and 8-hydroxy-2′-deoxyguanosine (8-OHdG) seem to be potential biomarkers. Biomarkers of patients with acute encephalitis and acute encephalopathy are screened in blood and serum [35]. Moreover, CSF PCR is highly useful to detect acute encephalitis in patients. Mainly biomarkers for brain injury are considered through a systematic screening of most vital and functional cells and tissues which are affected by neonatal encephalopathy [35]. However, different hosts show different viral tropisms and host immune responses prior to viral entry into the central nervous system. It is possible that it depends on antigen profile and protective strength of immune system. Other than damaging cells and tissues JE virus affect the glutamate aspartate transporter, glutamate transporter 1 and ceruloplasmin levels. There was observed an elevated level of LDH in the animals infested with wild strains of viruses [94].

Further, detection of N-glycosylation sites of JEV virus, E protein, and focal pathological effects such as focal neuronal degeneration with diffuse and focal microglial proliferation and lymphocyte perivascular cuffing strongly work as biomarker. Brain microvascular endothelial cells represent a functional barrier and could play an important role in leukocyte central nervous system trafficking [95]. Upon entry and infection of the CNS, these viruses can induce a rapid inflammatory response characterized by the infiltration of leukocytes into the brain parenchyma. Both chemokines and their receptors are involved in coordinating complex leukocyte trafficking patterns that regulate viral pathogenesis *in vivo*. However, key cellular events occur during the infection process and the immunodiagnostic role of these cells will become a strong future biomarker to identify the infiltrating virus in host cells [96].

## 7. Physiological Markers

Though it is very difficult to detect metabolic impairments in pathological cells, cell cultures of neuronal cells/nerve cells a very difficult task. If are possible these can be used for justifying virus generated physiological defects. It would also help to observe behavior of viral antigens *in vitro* to various nerve cell membrane molecules, neurotransmitters, ions and synaptic binding of inhibitory proteins. Such cell culture systems will certainly help to explore, cellular entry of virus its invasion and progression of disease and pathogenesis. However, measurement of acetylcholine activities in infested cells is still lacking. More exceptionally, the behavior of virus toxins to Na+K+ATPase pump in sensory and motor nerve fibres and proton deficiency can be correlated with pathogenesis. JEV infection caused increased intracellular ROS production and activation of ASK1-ERK/p38 MAPK signaling in human promonocyte cells [97]. Similarly, increased level of free radicals due to oxidation of bio-molecules in patients body also indicates higher neuronal damage/injury [98] that works as an important clinical biomarker of many viral infections [98]. However, maximum increased levels of ROS species (ROS), nitric oxide (NO), peroxinitite (OONO)(−) causes apoptotic cell death of neuronal cells. It also aids to generate acute JE with representative signs and symptoms of neuronal shrinkage and tissue necrosis [100]. Further, downregulation of thioredoxin, increased intracellular ROS and activation of ASK1/ERK/p38 MAPK signaling are associated with JEV induced apoptosis [97] (Yang et al.). However, thioredoxin prohibits JE pathogenesis by suppressing oxidative stress pathway [97]. Further, viral infection inhibited the expression of cell maturation surface markers (CD40, CD80, and CD83) and MHCl and impaired the ability of P3-infected DCs for activating allogenic naïve cells [100]. It impairs T-cell maturation, modulates cytokine productions and expanded regulatory T cells [97]. However, both structural and functional impairments occurs in neurons of infected patients if identified exactly could be used as important biomarkers to know virus generated pathogenesis.

However, other than damaging cells and tissues, JE virus affect the glutamate aspartate transporter, glutamate transporter 1 and ceruloplasmin levels. There was observed a rapid increase in total LDH level in the animals infested with wild strains of viruses that indicates severity of virus infection [94]. However, mice infected with wild strains of JE showed all five isoenzymes, among which LDH 1 disappeared after 12 days of infection but LDH 2 and LDH 3 persisted for 3 week, while WN virus stain showed an extra band near LDH 4 [94] Contrary to this attenuated strain of JE did not produce any change either of the total content of the enzyme or of the isoenzyme pattern. In the plasma of mice infected with wild strains of WN viruses, only 4 isoenzyme bands (LDH 2, 3, 4, 5) were detected in the gel. More important is LDH 3 persisted longer than LDH 2. Similarly, an alteration in serum sodium level, liver enzyme function and ADH secretion also mark JEV generated morbidity. Further, an elevation in aspartate aminotransferase (AST) and alanine aminotransferase (AMT) enzyme levels indicate virus inoculation in patients [101]; though, it cannot identify severity of Japanese encephalitis or its outcome. Similarly, almost no report is available on hormonal changes in JE patients those who have recovered. Certainly structural changes occur in endocrine glands, hormone deficiency and hypersecretions may correlate to the JEV generated effects. However, an instant increase in cellular enzyme mainly LDH and glucose transporters, and hormonal level could work as an important disease marker for JEV diagnosis in future. Hence, metabolic impairments are correlated to glucose utilization, then the severity of disease resulting in neurophysiological changes could be gauzed [62].

## 8. Immunohistochemical Biomarkers

Similarly, presence of viral antigens in tissue and cells also unravel neuropathogenesis caused by JEV. However, immunocytochemical localization of viral antigens by using immunofluorescence and immunohistochemical methods [102] helps to know structural changes caused by JE virus and works as an important biomarker (Table 1). Similarly, flow cytometry also helps to recognize viral invasion, cell death, and disease prevalence inside host [69]. However, detection of pathological changes by immunohistological methods in thalamus, substantia nigra, brain stem, hippocampus, cerebellum, and spinal cord by more clearly display the reasons of morbidity in JE patients. Similarly, pathological changes such as focal neuronal degeneration with diffuse and focal microglial proliferation and lymphocytic perivascular cuffing could also clarify cellular interactions of virus-secreted molecules serving as biomarkers. However, after establishing correlation between structural changes and cellular and tissue specific abnormalities, JE disease progression and status could be decided. Furthermore, for detection of JE progression and its related effects, neurosphere cultures are used [69]. Similarly, for fast analysis of JE virus antigens an indirect IgG immunofluorescence test (IIFT) is applied (Table 1). It is highly sensitive and specific method [102], which is used to detect antigen titers in the mouse brain mainly in hemisphere by confirming the binding of JEV antigen with rabbit anti-JEV serum. It is highly useful method for diagnosis of acutely infected persons and is a valuable alternative to the other established methods for detecting anti-JEV antibodies and humoral immune response after vaccination [6]. However, immunological relationships among flaviviruses can be established by detecting the level of MAbs in immunofluorescence and neutralization tests (Table 1) [103, 104].

JE virus targets neurons and generate neurotropism that persists in human cells, mainly in nerve and different blood cells (erythrocytes, lymphocytes, granulocytes, and monocytes). It is quite interesting that JEV could not replicate in erythrocytes, granulocytes or lymphocytes but it cultures in monocytes, because these cells support virus replication [105], but JEV could replicate more efficiently in neuroblastoma (HTB-11) cells than in monocytes after infection for 48 h. However, JEV-infected neuroblastoma cells suffered heavy cell apoptosis in 2 days and decreased viability to less than 1% in 5 days [105], while monocytes could take up JEV rapidly and display a log scale increase of intracellular JEV titers in 9 h after infection that prolonged for more than 3 weeks. Thus, JEV-infected monocytes play an important role in harboring JEV for eventual transmission to NB cells and modulation of JEV-induced NB cell apoptosis may be useful in treating patients with JE [105]. It is confirmed by expression of viral NS3 antigen and virus plaque-forming units.

More specifically, influenza virus is surrounded by an outer envelope, a lipid bilayer acquired from the plasma membrane of infected host cells during the process of budding. Besides this, two hemagglutinin (HA) and neuraminidase (NA) proteins form radiating projections inserted into the outer envelope. More specifically, hemagglutinin trimer binds to sialic acid groups on host cell glycoproteins and glycolipids by way of a conserved amino acid sequence that forms a small groove in hemagglutinin molecule [105]. While neuraminidase cleaves N-acetylneuramic acid from ascent viral glycoproteins on host cell membrane and facilitates viral budding from the infected host cells [105]. Similarly, glucosidase inhibitor of endoplasmic reticulum, blocks the trimming step of N-linked glycosylation, and helps to eliminate the production of several endoplasmic reticulum-budding viruses such as dengue type II (DEN-2) and JEV83.

Serum proteome, cytokines and inflammatory analysis of adults with primary dengue infection reveal predictive markers of DHF. These markers display three different stages of infection representing the early febrile, defervescence and convalescent stages. Using fluorescent bioplex assays, 27 cytokines were detected in serum samples of DHF infected patients. Additionally, multiple mass spectrometry and comparative analysis of serum proteome as well as measurements of protein adducts-3-nitrotyrosine and 3-chlorotyrosine as surrogate measures of free radical activity act as molecular marker for DHF [106]. Few immunological studies provide evidence that TLR2-MyD88 and p38 MAPK signal pathway might be involved in JEV-mediated inhibition of cross-presentation of soluble and cell-associated antigens. However, modulation of cross-presentation of exogenous antigens through TLR signaling has important implications for antiviral immune responses against JEV infection. It will help in will development of effective vaccination strategies [84].

In addition, the production of reactive oxygen species production and activation of ASK1-p38 MAPK signaling pathway might be associated with JEV NS2B-NS3 protease induced mitochondria. It mediate apoptosis in human medulloblastoma cells, and serve as an important biomarker for JEV. In addition, it might be useful in recognition of cellular and molecular pathogenic effects induced by JE virus infection [97]. It clearly shows that fatality of infected patient occurs due to extensive neuronal dysfunction rather than neuronal destruction in the CNS [58]. Further, oxidative damage also plays an important role in the pathogenesis of viral infections of the nervous system [98]. Further, for immunocytochemical localization of virus proteins (NS3), and cloning expression of NS3 genes [75] allow to detect neurovirulence generated by nonstructural JE virus proteins [75]. Similarly, western blot and immune-florescence analysis using the anti-NS3 antibody also explore effects of nonstructural proteins in human and experimental animals [75]. Thus, double immunostained cells with the anti-NS3 antibody and anti-flag antibody clearly show the presence of virus secreted antigens. However, virus structural proteins either recombinant or natural help in establishing the cause of infection and are considered as important protein markers of JE virus. Further, oxidative damage also plays an important role in the pathogenesis of viral infections of the nervous system [98].

## 9. Virus Proteins as Biomarkers

JEV contains positive single stranded RNA genome, approximately 11 kb in length. Virus genome contains a single open reading frame with a well-arranged gene order as 5′ C-prM-E-NS1-NS2A-NS2B-NS3-NS4A-2 K-NS4B-NS5 3′ [107, 108]. It encodes viral proteins [109, 110] mainly a precursor polyprotein having three structural proteins (C, prM, and E) and seven nonstructural proteins (NSI, NS2, NS2B, NS3, NS4A, NS4B, and NS5) [111]. Among nonstructural proteins, NS3 is a multifunctional protein having 619 amino acid residues and shows enzymatic activities like serine protease, helicase and nucleoside triphosphatase. NS3 plays important role in the processing of the viral precursor poly protein and the replication of viral genomic RNA [112]. In infected cells, NS3 is associated with microtubules and tumor susceptibility gene 101 protein, and plays essential role in viral packing, intracellular trafficking of various viral components. It was detected in the brain of JEV infected patient mainly in the cytoplasm of pyramidal neurons of the cerebrum [113], granule cells, small cells and Purkinje cells of the cerebellum after 12 h after-infection. The Purkinje cell of the cerebellum is one of the target cells of JEV infection [9]. NS3 is an important agent that generates neurovirulence in patients [114]. Suppressive effects, neurovirulence, and host immune responses generated by different JE viral proteins and antigens are mentioned in Table 1.

The flavivirus nonstructural glycoprotein NS1 is a cell surface protein (soluble entity) that generates neurovirulence in host neural cells after peripheral inoculation of virus, a multifunctional protein that shows mechanistic function [115] and assists the virus in neuronal invasion [116]. It plays important role in pathogenesis and cellular profusion and acts as a virulence determinant [117–119], and serves as a marker of Dengue virus infection in man [120] and mosquitoes [121]. Both secreted and cell-surface-associated NS1 are highly immunogenic and implicate disease pathogenesis [122–126]. This also occurs as a viral antigen and circulates in the sera of JE infected patients. It shows host immune response and elicits protective immunity in mice [127]. It plays an important role in establishment of pathogenesis and is used to generate protective antibodies against flavivirus [128–130]. It shows homology with dengue virus protein and its deformation affect CNS in mouse [131, 132]. Intracellular NS1 plays an essential cofactor role in virus replication and has been shown to colocalize with dsRNA and other components of replication complexes [133, 134]. It was found at different cellular locations either cell-membrane-associated (mNS1), in vesicular compartments within the cell, or on the cell surface, and as a secreted lipid-rich, extracellular (nonvirion) species (sNS1) [135, 136], or hexameric lipoparticle. [137–139]. More specifically, secreted form of NS1 shows wider interactions with host proteins and other bio-molecules and found implicated in immune evasion strategies and playing a direct role in pathogenesis. Therefore, NS1 functions as an important biomarker for early diagnosis of JE disease in infected hosts. However, interaction of NS1 related to its structure and trafficking within and from the infected cell, and its possible role in viral replication may have very high value in diagnostic applications.

In addition, two more structural proteins C and prM are also identified in JE virus by using cDNA analysis. These proteins contain glycosylation sites that show similarity with TBEV and WNV N-linked glycosylation site in prM or E protein and display protective potential. More specifically, in JE viruses, the prM protein contains one putative N-linked glycosylation site at N15E protein and another site at N154. If deletion occurs in the above site, it lead to a decrease in viral release [140–142]. Similarly, mutations occurred in envelope and coat protein work as real elements, of neurovirulence determinants in mice. It is proved by preparation and use of chimeric viruses [143]. For example, poliovirus infection is largely confined to a specific subpopulation of neuronal cells occur in human central nervous system and shows PV tropism and neurovirulence [144]. However, mutations generated in putative N-linked glycosylation sites in Japanese encephalitis virus premembrane (prM) and envelope protein(E) showed enhancement in protective potential [145]. Similarly, N-linked glycans of viral proteins play important role in modulating immune response in host cells [146]. These are also important for maintaining appropriate antigenic conformations, mainly neutralization epitopes that potentially alter the proteolytic susceptibility of these proteins [146, 147].

There is another major structural envelope protein E that contains numerous neutralization epitopes which play important role in viral attachment, membrane fusion and entry of virus into host cell. E protein also contain one putative N-linked glycosylation site at NS154 [132] that plays major role in determination of virulence phenotype. Its putative receptor binding domains induces the host immune response [148, 149]. E protein showed single amino acid substitutions, which are sufficient to cause loss of neurovirulence [150, 151]. Besides, E (envelope) protein NS3 is the main protein that is responsible for pathogenesis and show immune response. However, viral proteins NS3 protease in association of NS2B cofactor significantly induce higher degrees of apoptosis and trigger higher caspase 3 activities in human medulloblastoma cells [64]. Similar to NS1 viral proteins, E proteins are well-known targets of the protective antibody response against flavivirus infection and contain virulence determinants [128–130] (Table 1).

However, so many biological markers of neurovirulence have been identified [152] but molecular determinants of virus specific factors, which account for virulence, are still unknown. These might be highly specific, more confirmatory than biological markers because they are encoded with in multiple region of neurotropic RNA viruses [153–155]. It is also proved by single site mutations generated in the flavivirus genome, which encode envelope protein hinge region that resulted in a significant increase in virulence in mice and monkeys [156]. Mutations that occur in structural and nonstructural viral proteins are responsible for generation of neurovirulence. These might be of reversion or deletion type. Similarly, mutations occurred in matrix (M) protein of vesicular stomatitis virus (VSV) generate neurovirulence. These M protein mutants of VSV can be used as vaccine vectors [157]. Interestingly, measles virus also shows very high neuroinvasivness in animal models but shows limiting neurovirulence in humans. Similarly, poliovirus causes very high susceptibility to CNS and grows in neural cells but shows a limited neurovirulence in host. Furthermore, serial passages done for yellow fever virus (YF 17D) in mouse brain enhances neurovirulence and causes a reduction in survival time after intracerebral inoculation of experimental mice [158]. However, severity of infection increases neurovirulence, which may occur due to virus invasion in nerve cells mainly in brain. It also shows inflection of virus in spreading route, mainly in neuroaxis, brain, and spinal cord [159]. More exceptionally, an adjacent stem loop structures identified within the JRES and internal ribosomal entry site cooperatively determine neuropathogenicity [144]. However, weaker interactions occurred between virus antigens and host immune cells generate chances of more neuronal invasion by virus that may lead to high pathogenesis [160]. In such a condition, both cellular and humoral responses cannot strike well upon virus and generated molecules, and even the body's own infected cells [161]. However, candidate molecules, which are secreted after invasion of virus by host body cells can be, used as good biomarkers for neurovirulence determination [162].

Recently, microarray researches cleared that neurons can make their own defense against Japanese encephalitis viral infection even that they do not show power of regeneration. It is a very challenging job because neurons are immunologically quiescent and an improvement in proinflammatory effects is very difficult task for immune-mediated control of viral infection and repairing of neuronal injury. If it will become possible to have some novel inducers of neuronal regeneration, it will be a land mark step for developing strategies for limiting the severity of CNS disease mainly pain, inflammation and neurological impairments in patients. However, monocyte and macrophage receptor CLEC5A they are found involved in severe inflammatory response in JEV infection could be sloweddown.

Similarly, identification of different protein functions of structural and nonstructural proteins of JEV genome may also facilitate a mechanistic understanding of Japanese encephalitis virus (JEV) infection. However, protein functions common to both structural and nonstructural proteins such as iron-binding, metal-binding, lipid-binding, copper-binding, transmembrane, outer membrane, channels/pores pore-forming toxins (proteins and peptides) could work as important biomarkers. Similarly, nonstructural proteins perform functions like actin binding, zinc-binding, calcium-binding, hydrolases, carbon-oxygen lyases, P-type ATPase, proteins belonging to major facilitator family (MFS), secreting main terminal branch (MTB) family, phosphotransfer-driven group translocators and ATP-binding cassette (ABC) family group of proteins could also establish diagnostic facts about JEV and are considered as important future biomarkers. Most flavivirus nonstructural (NS) proteins correlate with virus-induced inflammation and immune escape. However structural proteins besides belonging to same structural group of proteins (capsid, structural, and envelope), they also perform functions like nuclear receptor, antibiotic resistance, RNA-binding, DNA-binding, magnesium-binding, isomerase (intramolecular), oxidoreductase and participate in type II (general) secretory pathway (IISP) [163]. It will also help to develop new drugs.

JEV protein E shows a potential to induce antiviral responses by synthesizing anti-JEV neutralization antibodies. However, E protein derived peptides also contain virus neutralization epitopes, which assist in generation of JEV-neutralizing antibodies [164]. Similarly, plasmid encoding Japanese encephalitis virus premembrane and envelop genes generate immune responses and induction of protective immunity against Japanese encephalitis in mice [15]. Following an immune response these also induce specific memory B-cells and long lasting antibodies in animal hosts [165]. Similarly, membrane anchored and secretory envelope proteins elicit immune responses in experimental animals [14]. However, it is established that plasmid encoding Japanese encephalitis virus protein induce neutralizing antibody or cytotoxic T-lymphocytes in mice [16, 17]. Further, neutralization ligands selected from Phage displayed librarian mimic the conformational epitope on domain III of the Japanese encephalitis virus envelop protein [166]. Similarly, human C virus envelop protein E1 contains N-glycosylation sites and enhances specific cellular and humoral immune response [167].

More specifically, interferon (IFN) antagonists of Japanese encephalitis virus (JEV) proteins contribute to the JE pathogenesis [168]. However, NS4A proteins of West Nile virus and dengue type 2 virus demonstrated inhibition of IFN signaling. Similarly, JEV NS4A without the C-terminal 2 K domain partially blocks activation of an IFN-stimulated response element (ISRE)-based cis-reporter by IFN-alpha/beta. It also significantly inhibits the phosphorylation levels of STAT1 and STAT2, but not TYK2 in the IFN-treated cells [168]. Moreover, the N-terminus of an RNA helicase DDX42 protein specifically binds to JEV NS4A *in vitro *and such interaction is localized in human medulloblastoma TE-671 cells by confocal microscopy. Importantly, the expression of N-terminal DDX42 is able to overcome JEV-induced antagonism of IFN responses [168]. However, the level of IFN alpha and beta works as important disease marker of JE virus infection.

However, chimeric yellow fever (YF) virus/Japanese encephalitis (JE) virus vaccine (ChimeriVax-JE) constructed by insertion of the prM-E genes from the attenuated JE virus SA14-14-2 vaccine strain into a full-length cDNA clone of YF 17D virus [169] induces protective immunity against JE virus after immunization. Moreover, single site mutations done at E279 position were located in a betasheet in the hinge region of the E protein that is responsible for a pH-dependent conformational change during virus penetration from the endosome into the cytoplasm of the infected cell [169]. After intracerebral inoculation, the E279 Lys virus was restricted to extraneural replication in monkeys, as viremia and antibody levels were significantly reduced compared to those for the E279 Met virus [169]. More exceptionally, it shows a reduced viscerotropism in humans in comparison to mice [169]. However, new antigenic sites generated by using site directed mutagenesis in virus genome and expression of these mutated virus genes could help to mark the negative and positive effects of prM genes in experimental animals and proved to be good biomarkers for identifying JEV generated pathogenesis.

Furthermore, Japanese encephalitis virus (JEV) is also detected in clinical samples by using one step TaqMan reverse transcription polymerase chain reaction (RT-PCR) (Table 1). It is highly sensitive, specific, rapid and quantitative diagnostic method used for the fast detection of JEV in laboratory and field collected samples [170]. It helps in the quantification of JEV which is accomplished by preparing a standard curve plotting cycle threshold values (C(t)) versus infectivity titer [170]. It is used for detection and quantification of JEV and its RNA genome in plasma samples in 2 days after inoculation experimental animals with KV1899 strain [170]. This test could also help to establish strain specific variants of RNA genome, which will work as perfect biomarkers at the molecular level for exploring alternate reasons of JE-induced neuropathogenesis and disease.

## 10. Molecular Markers

Certain biomarkers, which can display role of genes in disease pathogenesis and mutations, evolved and its expression profiles could explore the disease status and morbidity related effects. These biomarkers can be identified by using basic and acceptable techniques used in genomics and proteomics of JE virus. However, genomic approaches such as northern blots, SAGE, DNA micro array are used to find disease-specific candidates related to gene structure and function. Similarly, regular protein profile obtained from an infected and uninfected patient could be obtained by analyzing body fluid, tissue and cells on 2D PAGE, LC-MS, SELDI-TOF or (MALDI-TOF), antibody microarray and tissue microarray could establish changes related to structural and nonstructural protein in human hosts. Moreover, all structural and nonstructural protein changes occur in patients at different periods could clearly establish morbidity caused by Japanese encephalitis virus. Further, genome-specific markers help to detect JE virus mutations occurred in ecologically adapted antibody resistant strains. Other tests, which are used, for detection of virus are reverse transcription PCR (RTPCR) and electron microarray which are also used to establish presence of JEV in clinical samples (Table 1). Therefore, for a quick start in treatment a confirmed diagnosis of JE based on rapid immunodiagnostic tests is essentially required.

Similarly, mutation analysis of virus proteins and its interactions to disease marker genes could make JEV diagnosis much easier. However, statistical analysis of the envelope gene and prM region of JEV virus could find significant variations in nucleotide sequences. Further, presence of selective forces acting on these regions investigated by computing the relative rate of synonymous substitutions could explore heterogeneous genotypes circulating in endemic population [171]. It could work as an important biomarker. Estimates of mean of nucleotide distances for different region of the E gene could establish the divergence occurred in flavivirus and can present possible divergence in future strains. However, comparative analysis of complete genome sequence and its full-length sequence based phylogenetic analysis could confirm the particular JEV strain belonging to particular genotype. However, molecular substitutions per site could explain the role of distantly placed viruses and its possible neurovirulence in human host. If compared the polyprotein as a whole, then unique difference in amino acid substitutions could be achieved, it will help to know the functional differences in a newly formed protein and its workable antigenicity. However, either these old and new functional differences created or natural one could suggest all the possible modifications in the epitopes. It will work as an strong confirmatory marker for determination of level of morbidity in different JE virus strains circulating in the endemic area. DNAzyme mediated inhibition of virus replication is an important molecular marker to find presence of neurotropic virus titers inside host. This oligonucleotide mediated inhibition also work as drug biomarker for JEV [172].

Moreover, a molecular analysis of transcriptomic data of JE virus could ably find exact genotype and its generated pathogenesis in human hosts. Therefore, cloned genes can be transfected into cultured cells and examination of tissue specific gene expression and its comparison in different cells may provide overall information at gene level functions and its alternations more accurately. Further, identification of candidate host gene and systematic mRNA profiling could establish real cause of JEV pathogenesis. Moreover, microarray analysis of mRNA expression profiles in spleen and brain could explore JEV infection and virus induced cellular and molecular changes in experimental animals and human hosts [173]. These circulating viral microRNAs have been reported as potential biomarkers for the neuroinvasive diagnosis of virus infection [174]. These are useful for diagnosis of viral infections since viral microRNAs should be released in the extracellular space after the death of infected host cells [175]. Interestingly, viral microRNAs in body fluids, varies from host to host may serve as specific markers for viral infection and disease progression or for therapeutic monitoring and drug development [175–177]. It is well known that significant pathways involved in differentially expressing genes are involved in cytokine-cytokine receptor interactions, natural killer cell mediated cytotoxicity, antigen processing and presentation, MAPK signaling and toll-like receptor signaling. However, these could work as multidimensional biomarker, which can make a clear picture of various biological processes and its related secretory molecules particularly comparing a large data set from DNA gene expression micro-array analysis from different JEV infected patients.

However, to emphasize the effects of individual virus genes and cluster of genes its expression level could strengthen the role of multiple genes in establishment of JE disease. However, multivariate functional genomic data could tell about time bound assimilation of new mutations and induction of pathogenic features in different hosts though which attain wider neurovirulence. However, covariance parameters of single and multiple gene functions could establish multiprocess pathways and variability across individuals. In addition, analysis of time course of gene expression data could explain temporal shifts in gene arrangement due to substitution mutations. However, most distinguished impact of gene on protein related variations and its best possible host responses will be known. These may be used as strong molecular biomarker for future.

Other strong markers could be obtained from metabolomics, lipidomics, glycomics, and secretomics studies. These are the most commonly used techniques, which identify metabolic pathways involved during virus cycle inside host body mainly pathways followed for generation of pathogenesis. However, utilization of metabolic components, combustion and end product formation could help to assess the disease status partially. Further, analysis of lipids provides unique physical properties of certain lipids in disease state. However, improvements in new analytical platforms have made it possible to identify and to quantify most of lipids metabolites from a single sample. However, parameters related to carbohydrate, lipid and protein profiling on mass spectrometry, chromatography, and nuclear magnetic resonance can be used as a marker for JE diseases. Mass spectrometry is used to delineate the relative concentration and composition of high-density lipoproteins (HDL) particles from lipid extracts isolated from JE patients and healthy volunteers could express some specific changes. However, levels of HDL, sphingomyelin, phosphatidylcholine, triglycerides and cholesterol esters could assign involvement of some specific pathway more suitable for virus invasion. Similarly, presence of certain metabolites in blood and urine are well-known biomarkers for influenza virus and *Staphylococcus aureus *coinfection [178].

## 11. Therapeutic Markers

Therapeutic markers could establish specific interactions of drugs to virus and host body molecules and cells. If these therapeutic effects are correlated, pathways of disease occurrence, morbidity status and clinical care targets could be decided in JE patients. However, there is an utmost need of chemotherapeutic agents, which could slow down the virus multiplication and cell invasion and neurotropism. These should inhibit formation of virus structural and nonstructural envelope proteins and can suppress the lethal action of virus generated molecules. Furthermore, action route of plant origin anti-viral components and its inhibitory effects must be investigated on virus genome, genes and proteins. Moreover, virus specific chemotherapeutic agents could also manage inhibition of virus multiplication that may result in inhibition of infection at an earlier stage. Moreover, herbal therapeutic agents, who can neutralize the virus-generated effects and show sustainable neuroprotective effect, are to be essentially explored. These novel molecules should possess enough potential to decrease the viral load; activate caspase-3 activity, reactive oxygen and reactive nitrogen species, microgliosis and proinflammatory cytokines in JE infected patients [179]. Interestingly, treatment with arctigenin improves the overall stresses caused by JEV and behavioral changes occurred in JE patients [179]. It shows antiviral, neuroprotective, anti-inflammatory and anti-oxidative effects and much successfully reduced the severity of disease induced by JEV [179]. Similarly, inhibition of ubiquitin-proteasome system by curcumin causes reduction in infective viral particle production from previously infected neuroblastoma cells [180]. Moreover, bioorganic compounds, which can significantly cut down virus generated cellular and tissue stress and injuries and might show repairing capacity will be on high agenda. More exceptionally, therapeutic molecules, which can stop virus lethal challenge, virus invasion in neuronal cells, and to diffuse the virus load in the patient and show quick solubilizing anti-inflammatory effects and restore behavioral impairments in JE patients, are to be highly required and explored. Minocycline is found to be broadly protective in neurological disease, which mainly reduce, inflammation, cell death and abrogated the effects by reducing blood-brain barrier damage [92]. It significantly reduces microglial activation, inhibits caspase 3 induction, and viral replication following Japanese encephalitis [68]. Similarly, few lectin molecules such as collectins, ficolins [181] and selectins [182] play important role in generation of innate defense acute phase proteins [46] that finish the infection [183]. Similarly, mild hypothermia therapy in children helps to reduce brain edema [184].

In addition, other strategies like RNA silencing and interference, activation of complement system are used to protect from JE virus infection. Similarly, a short hairpin RNA or lipid complexed small interfering RNA (siRNA) is used for RNA interference before virus challenge the immune system [185]. It also suppresses fatal encephalitis generated by two different flaviviruses. However, use of various types of cytokines, complement proteins, enzymes, antibodies and passive transfer of activated CTLs, T cells, B cells and NK cells can be used to destruct viral infection. Further, gases like nitric oxide (NO) have been shown to suppress Japanese encephalitis virus (JEV) RNA synthesis, viral protein accumulation, and virus release from infected cells [186, 187].

Further, to check the skipping and mutating behavior of virus cross-protective vaccines are to be prepared because new heterologous genotypes of JEV are emerging in endemic areas. Hence, new vaccines will be required to elicit protective levels of neutralizing antibodies against heterologous strains of genotype I-IV [188]. In addition, novel viral antigens from structural proteins or its derived peptides may be used for preparation of strong vaccines that can induce an overall anti-viral state by generating potential immune responses against JE virus [189, 190]. Similarly, a synthetic oligonucleotide-based DNAzyme significantly inhibit JEV virus replication and proliferation of Japanese encephalitis virus mouse brain and *in vitro* cell culture [171]. It also protects JEV-infected mice from death. It results in a sharp reduction in JEV titer in host brain, which may lead to an extended lifespan, or recovery of infected patient. Further, diverse mimotopes of active virus antigens that can mimic the JEV neutralizing antigen activity can be generated. Similarly, VLPs and fusion proteins are also used for generation of potential vaccines. Furthermore, so many JEV infectious mutant clones can be generated by insertion of short introns or cloning into artificial chromosomal systems [191]. However, for better treatment conserved sequence of all different types of structural proteins of JE virus that might challenge acute viral infections with multiple of overlapping clinical symptoms should be used for making multivalent vaccines. Further, occurrence of climate induced genotypic variations or mutations and other molecular changes occurred in flaviviruses must be identified, inexcusably added and considered for generating new potential vaccines. Furthermore, therapeutic could assign a significant effect on virus antigenic sites and more competitively bind to it. However, therapeutic biomarkers can explore the anti-viral potential of vaccines, post vaccination effects, seroconversion rate and suppressive effects of drugs on neurovirulence. However, for successful control of JE clinically reliable tests are to be used for proper diagnosis of JE virus in clinical samples with strong prophylactic and therapeutic measures. Recently whole genome microarray research tried to make neurons to make their own defense against Japanese encephalitis viral infection. These could work as future regenerative therapeutic markers for JE disease.

## 12. Conclusion

Unfortunately, due to lack of potential biomarkers for JEV detection and unavailability of timely treatment very high mortality is occurring almost every year in Southeast Asia. Because of shorter incubation period, high multiplication and infection rate, slow diagnosis, and unavailability of timely treatment high mortality is seen in different parts of India and its neighboring countries [192, 193]. Further, due to demographic and cultural reasons JE is regularly spreading in non-endemic areas. Recently, indigenous transmission of JEV is also observed in urban areas [194]. It is fact that in rural areas, no JE diagnostic facilities are available to confirm the disease and in most of the cases due to lack of strong confirmatory biomarkers, patients die with out having any therapeutic treatment. It has lead to an unexpected increase in the morbidity and mortality rates in rural pockets of India. Hence, there is an utmost need to have strong clinical biomarkers to decide the JE disease very fast at an earlier stage. Further, potential biomarkers are essentially required for deciding cause of disease, vaccination and monitoring the efficacy of therapies. Thus, by adopting rapid and proper diagnostic tests, one can improve the case detection rate; clinical index of suspects, difference between affected and non-affected people.

Further, genotype based neurovirulence, antigenicity, pathogenesis and mortality must be re-investigated by using new candidate markers, which can assist in exploration of pathological mechanisms followed by virus during invasion, persistence and clearance. More specifically in absence of potential biomarkers many facts regarding JE virus neuropathogenesis and other disease related effects are either undisclosed or incomplete. Further, for elimination of infectious viruses like JE, antigenic modifications are to be required to generate more appropriate and highly successful vaccines. It will necessitate antigenic profiling of all JE viruses and associating strains to explore the vary reasons of severity of epidemics. However, standardization of biomarkers will help in identification of new JEV mutant strains exist in endemic and JEV prone areas. It will also help in risk assessment, immunization and postvaccination success and immune effects in animals and human population to design new safe vaccines. A rapid test, that delivers a quick result should be followed. It will make possible for the physician to discuss with the patient how to proceed and if necessary to start treatment immediately after the test. In addition, strong confirmatory biomarkers will enable physicians to develop individualized treatment plans that will help in early primary care of JE patients. Conclusively, with aid of a perfect biomarker, clinicians will find a clear solution for progression and type of treatment required for JE patients. Naturally, the detection method for a biomarker must be accurate and as easy to be carried out as possible. The results from different laboratories may not differ significantly from each other, and the biomarker must naturally have proven its effectiveness for the diagnosis, prognosis, and risk assessment of the affected diseases in independent studies. A biomarker for clinical use needs good sensitivity and specificity. It should put a positive predictive value rather than negative for behavioral care of patient. Finally, all JE viruses related effects must be correlated with clinical studies, biomarker information and autopsy outcomes for knowing the last stage of severity of disease and failure of medicare.

Further, studies on core candidate markers for JE disease will characterize some more specific unknown facts about pathological mechanisms of marker regulation and expression. No doubt these will be more differentiated and complex than current mechanisms. These will provide a clear insight into various levels of JE virus pathogenesis and multidimensional diagnosis based on upgraded technological methods. There is another possibility that neuro-viral infections may invite some coinfections and that may cause clinical complications. Therefore, it is possible that a protozoan or a bacterial infection will also infect the patients during the progression of JE disease. However, in a state of disease outbreak by drug resistant microbe and antibody resistant JE virus strain highly specific biomarkers would be required to validate the cause of infection. However, advent of new JEV markers could benefit clinician to find more exact and authentic reasons of risk progression and its management by applying various therapeutic measures against JE disease. Moreover, disease-related biomarkers should provide an indication about cause of disease, probable effect of virus and therapeutic treatment on patient. More specifically, both risk indicator and diagnostic biomarkers will make a balance between intensity of infection and possible dose required for the treatment. Furthermore, no doubt predictive biomarkers will provide a real assessment of most likely response of virus and host to a particular treatment type, while prognostic markers may clear the progression of disease with or without treatment. Similarly, therapeutic biomarkers may correlate drug-related and vaccine-related effects in patients. These will find exact drug response and rate of improvement and may limit the drug generated toxic/adverse effects due to prescription of over or low dose of any therapeutic agent in patients.

Therefore, collaborative research efforts are required both from industrial research institutions and from virology peer groups to develop some new innovative biomarkers for fast detection of JE virus in clinical samples. There must be a better understanding among clinicians, molecular and cellular neurobiologists, immunologists, for starting new research initiatives to make landmark innovations in the field of biomarker researches. Further, regenerative biomarkers would be developed for wound healing of virus injured neurons in CNS diseases. This is only possible by making fine coordination between interdisciplinary research fields mainly cell biology, molecular biology, immunology, virology and biomedical sciences. Further, various socioeconomic and medico-economic acceptable models of must be designed and used to work out JE risk stratification in endemic population. For streamlining valuable insights innovative researches to maximize subject resources, data acquisition and multifaceted analysis should be of high priority. No doubt, biomarker studies could explore future perspectives of clinical diagnosis of JEV infected acute encephalitis patients and give rise new constraints for successful JE control in future. Hence, biomarkers that could help in early diagnosis, disease prevention, drug target identification, drug response are to be needed for management of JE disease. It will provide a better safe guard to pediatric groups, in providing strong preventive and therapeutic measures. For this purpose, JEV diagnostic laboratories must be established in endemic areas for timely diagnosis of JE virus to avoid extra delay in treatment. It will certainly cut down mortality rate in infants. Further, time bound consistent powerful surveillance of JEV in endemic areas will provide a real time data on its infectivity, morbidity, mortality and clinical care of patients. In addition, more stress must be given on interventions like mosquito control and avoidance of human exposure to mosquitoes and reservoir hosts can make successful control of JE disease. Therefore, strong prophylactic and therapeutic measures are required for successful control of JE disease in endemic areas. More specifically, biomarker studies may lead to some unexpected results unknown multidimensional which can provide a clear insight on pathophysiology of JE virus disease in the future. Hence, strong recommendations are being made to improve biomarkers used in clinical practice, and more sophisticated new emerging biomarkers are to be generated and included in clinical diagnosis of JEV infected acute encephalitis patients and will also give rise to new constraints for successful JE control.

## Figures and Tables

**Table 1:** Different JEV specific tests for clinical diagnosis of Japanese encephalitis virus in body fluid, cells, and tissues of patients.

Method	Sensitivity	Confirmatory diagnosis
IgM capture ELISA (enzyme linked immunosorbent assay) DEIA dipstick enzyme linked immunosorbent assay Panbio JE-DEN IgM Combo ELISA JE-Chex IgM capture ELISA	Highly sensitive and confirmatory Sample 5 *μ*L, total time 135 minutes, antigen coated plate HRP and tetramethyl benzidine (substrate) Highly sensitive and confirmatory Detection of JEV positive and negative results in CSF and serum	Can differentiate infection type, intensity, and presence of JEV strains and detects specific IgM in the cerebrospinal fluid or in the blood Detection of immunoglobulin in human serum to JEV- derived antigens. *In vitro* diagnostic use Status of viral encephalitis, neuroinflammation
MAC-ELISA	MAC ELISA is used to diagnose secondary flavivirus infection	Used to detect true positive and true negative sensitivity and specificity in JEV affected patients
Single TaqMan assay	Highly sensitive and confirmatory	Diagnoses virus antigens

Immunofluorescent test	Highly sensitive and confirmatory	Works as a valuable alternative to the established methods in detecting anti-JEV antibodies after vaccination in travelers and helps in the diagnosis of acutely infected persons, *in vitro* labeling of NPC cells
Fluoresecent markers are used Fluorescent dye 7-ADD binds to DNA. The labelled cells are detected by FL-1 channel by FACS and are analyzed by using Cell Quest Pro software to quantify percentage of labelled cells.	The amount of flouresecent antibody bound to each cell can be quantified	
Florescence resonance energy transfer (FRET)	Highly sensitive	Detects interaction of antigens in cells

Plaque reduction or JEV-antibody neutralization test (PRNT)	Moderately sensitive and confirmatory On incubation, the antibody forming cells release immunoglobulin which coats the surrounding erythrocytes. Complement causes lysis of coated cells and plaque clear or red cells are counted. Hemolytic efficiency of IgM antibodies is detected	Can detect humeral immune response generated after immunization with JE inactivated vaccine Used for viremia determination Percentage neutralization is calculated from number of plaques obtained
Microcomplement fixation test	Moderately sensitive	Can detect cellular factors and antigens
Virus overlay protein binding assay (VOPBA)	Highly sensitive and confirmatory	Detects JE virus receptor molecules on the cells
YUNEL assay	Highly sensitive	Apoptosis, cell membrane disruption, and morphology

Lumbar puncture test and CSF analysis	Moderately sensitive and confirmatory	Probable and confirmed JE

MRI (magnetic resonance imaging)	Moderately sensitive and confirmatory	Can locate bilateral thalamic lesions with hemorrhage, and any abnormality generated in basal ganglia, putamen, pons, spinal cord, and cerebellum may also show pathological abnormalities

CT scan (computed tomography)	Highly sensitive and confirmatory	Can locate hyperintense lesions in the areas of the thalamus, cerebrum, and cerebellum

EEG (electroencephalogram)	Moderately sensitive and confirmatory	Reveals diffuse and burst suppression

CBC (complete blood count)	Confirms the presence of JEV infection in children and helps in clinical analysis of blood parameters	Detect leukocytosis, leucopenia, anemia and thrombocytopenia, and supportive lymphocytic pleocytosis

Platelet count	Sensitive and supportive for clinical analysis	Can detect effect of fever on blood platlets

Hemagglutination inhibition test (HA)	Moderately sensitive Agglutination is done by using antigen coated particles	Antibody detection to detect rheumatoid factors identification of antibodies to soluble antigens. HA is used to detect JEV in various passages

Compliment fixation test (CF) or crosslinking of antigens	Moderately sensitive	Antibody detection. Surface antigens are detected by using labeled antibodies. Both monovalent and divalent antibodies are used
Immunotyping	Highly sensitive and confirmatory	Differentiates genotypes of JE virus

RPHA, IFA, immunoperoxidase	Moderately sensitive	Antigen detection

Immunoblotting	Highly sensitive	JEV generated infection in NCPs and recognizes decrease in the number of colony forming neurosphere and their self-reveal, HRP, PBS-T

IDD (immunodouble diffusion test)	Moderately sensitive	Immunologic relationship between the antigens related or indicative or unrelated Precipitate forms an opaque line in the cross-reactive region

Cell death assay (annexin-propidium iodide staining test)	Highly sensitive	Can recognize apoptotic cell death in control and JEV infected cells. FITC labeled annexin and propidium iodide are used

Nephrometry	Moderately sensitive	Antigen and antibody dilutions are used to create cloudiness, and greater sensitivity can be generated by using monochromatic light from a laser and by adding PEG to solution to increase the size of aggregation

Neutralization tests	Moderately sensitive	Neutralization antibody titre in sera and in CSF can recognize homologous virus, the challenge virus, and the selected wild-type JE virus

Flow cytometry (FACS)	Highly sensitive	Intracellular signaling of JEV antigen, to detect percentage of anti-JEV-FITC positive cells.

Immunohistochemistry	Highly sensitive	Intracellular localization of NS3 by using anti- JEV antibodies

Precipitin test	Moderately sensitive	Quantitative analysis of antigen and antibody interaction

SRID	Highly sensitive	To know the amount of antigen in unknown samples

Neurovirulence test	Highly sensitive	To detect histopathological recognition of JEV pathogenesis in brain and in associated tissues. Prediction of level and cause of neurovirulence

Anticomplementarity Test	Highly sensitive	Identification of lesion scores

Hemolysin test	Moderately sensitive	Percent of hemolysis in RBCs

DNA microarray	Highly sensitive and confirmatory	Expression of genes and proteins

Site directed mutagenesis	Highly confirmatory	Detects amino acid substitutions in E, NS1, and NS2 proteins, clone-specific substitutions, and heterogeneity substitutions and is used to detect possible mutations in structural and non structural viral proteins

Real-time polymerase chain reaction (RT-PCR)	Highly sensitive	Target sequences can be detected in genes and viral genome. Amplification of immunotype strain, cloning, and expression of NS3 gene of NS3 protein of JEV

RNA studies oligonucleotide primer-based detection of JEV functional sequences in different genes and gene copies	Highly sensitive	Detects molecular pathogenesis at the level of enzymes, genes, factors, and proteins. Synthesis and secretion of JEV-induced proteins

*Presence of JEV viral and virus secreted antibodies are detected in cerebrospinal fluid (CSF) and serum samples. For component-based detection of JEV, a wide variety of conventional techniques such as viral neutralization, hemagglutination (HI), and complement fixation and immunoflourescent staining are used. Laboratory diagnosis of JE virus is mostly confirmed by immunological, molecular, and biophysical methods. Most of the laboratory-based tests and clinical diagnostic tests are routinely used to detect presence of JEV virus and its pathogenesis but all such tests are labor-intensive, expensive, and cumbersome.

## Abbreviations

NS: Nonstructural proteins
CSF: Cerebrospinal fluid
VSV: Vesicular stomatitis virus
JEV: Japanese encephalitis virus
TCR: T-cell receptor
CTL: Cytotoxic Lymphocytes
NCPs: Neural Progenitor Cells
CNS: Central nervous system.

## References

[1] Kabilan L, Rajendran R, Arunachalam N (2004). Japanese encephalitis in India: an overview. *Indian Journal of Pediatrics*.

[2] Gunakasem P, Chantrasri C, Simasathien P, Chaiyanun S, Jatanasen S, Pariyanonth A (1981). Surveillance of Japanese encephalitis cases in Thailand. *The Southeast Asian Journal of Tropical Medicine and Public Health*.

[3] Cardosa MJ, Choo BH, Zuraini I (1991). A serological study of Japanese encephalitis virus infections in northern Peninsular Malaysia. *The Southeast Asian Journal of Tropical Medicine and Public Health*.

[4] Desai A, Ravi V, Chandramuki A, Gourie-Devi M (1994). Detection of neutralizing antibodies to Japanese encephalitis virus in the cerebrospinal fluid using a rapid microneutralization test. *Serodiagnosis and Immunotherapy in Infectious Disease*.

[5] Shrivastva A, Tripathi N, Parida M, Dash P, Jana A, Lakshmana Rao P (2008). Comparison of a dipstick enzyme-linked immunosorbent assay with commercial assays for detection of Japanese encephalitis virus-specific IgM antibodies. *Journal of Postgraduate Medicine*.

[6] Litzba N, Klade CS, Lederer S, Niedrig M (2010). Evaluation of serological diagnostic test systems assessing the immune response to Japanese encephalitis vaccination. *PLoS Neglected Tropical Diseases*.

[7] Heinz FX, Stiasny K (2012). Flavivirus and their antigenic structure. *Journal of Clinical Virology*.

[8] Solomon T, Thao LTT, Dung NM (1998). Rapid diagnosis of Japanese encephalitis by using an immunoglobulin M dot enzyme immunoassay. *Journal of Clinical Microbiology*.

[9] Murata R, Hashiguchi K, Yoshii K (2011). Seroprevalence of West Nile virus in wild birds in Far Eastern Russia using a focus reduction neutralization test. *American Journal of Tropical Medicine and Hygiene*.

[10] Zhou W, Pool V, Iskander JK (2003). Surveillance for safety after immunization: vaccine Adverse Event Reporting System (VAERS)—United States, 1991–2001. *MMWR. Surveillance Summaries*.

[11] Zhang S-B, Li P, Liu X-Z (2010). Analysis on neutralization antibody titer of Japanese B encephalitis virus in healthy population in Shaanxi province. *Zhongguo Yi Miao He Mian Yi*.

[12] Saini M, Vrati S (2003). A Japanese encephalitis virus peptide present on Johnson grass mosaic virus-like particles induces virus-neutralizing antibodies and protects mice against lethal challenge. *Journal of Virology*.

[13] Wang K, Deubel V (2011). Mice with different susceptibility to Japanese encephalitis virus infection show selective neutralizing antibody response and myeloid cell infectivity. *PLoS ONE*.

[14] Kaur R, Sachdeva G, Vrati S (2002). Plasmid DNA immunization against Japanese encephalitis virus: immunogenicity of membrane-anchored and secretory envelope protein. *Journal of Infectious Diseases*.

[15] Konishi E, Yamaoka M, Khin-Sane-Win K-S, Kurane I, Mason PW (1998). Induction of protective immunity against japanese encephalitis in mice by immunization with a plasmid encoding Japanese encephalitis virus premembrane and envelope genes. *Journal of Virology*.

[16] Konishi E, Ajiro N, Nukuzuma C, Mason PW, Kurane I (2003). Comparison of protective efficacies of plasmid DNAs encoding Japanese encephalitis virus proteins that induce neutralizing antibody or cytotoxic T lymphocytes in mice. *Vaccine*.

[17] Marucci AA, Fuller TC (1971). Quantitative micro-complement fixation test. *Applied Microbiology*.

[18] Okuno T, Okada T, Kondo A, Suzuki M, Kobayashi M, Oya A (1968). Immunotyping of different strains of Japanese encephalitis virus by antibody-absorption, haemagglutination-inhibition and complement-fixation tests. *Bulletin of the World Health Organization*.

[19] Takegami T, Miyamoto H, Nakamura H, Yasui K (1982). Differences in biological activity of the V3 envelope protein of two Japanese encephalitis virus strains. *Acta Virologica*.

[20] Kimura-Kuroda J, Ichikawa M, Ogata A, Nagashima K, Yasui K (1993). Specific tropism of Japanese encephalitis virus for developing neurons in primary rat brain culture. *Archives of Virology*.

[21] Heinz FX, Berger R, Tuma W, Kunz C (1983). A topological and functional model of epitopes on the structural glycoprotein of tick-borne encephalitis virus defined by monoclonal antibodies. *Virology*.

[22] Heinz FX (2012). Flaviviruses and their antigenic structure. *Journal of Clinical Virology*.

[23] Mathews JH, Roehrig JT (1984). Elucidation of the topography and determination of the protective epitopes on the E glycoprotein of Saint Louis encephalitis virus by passive transfer with monoclonal antibodies. *Journal of Immunology*.

[24] Roehrig JT, Mathews JH, Trent DW (1983). Identification of epitopes on the E glycoprotein of Saint Louis encephalitis virus using monoclonal antibodies. *Virology*.

[25] Schlesinger JJ, Brandriss MW (1983). 17D yellow fever virus infection of P388D1 cells mediated by monoclonal antibodies: properties of the macrophage Fc receptor. *Journal of General Virology*.

[26] Peiris JSM, Porterfield JS, Roehrig JT (1982). Monoclonal antibodies against the flavivirus West Nile. *Journal of General Virology*.

[27] Henchal EA, Gentry MK, McCown JM, Brandt WE (1982). Dengue virus-specific and flavivirus group determinants identified with monoclonal antibodies by indirect immunofluorescence. *American Journal of Tropical Medicine and Hygiene*.

[28] Bell JR, Kinney RM, Trent DW (1985). Amino-terminal amino acid sequences of structural proteins of three flaviviruses. *Virology*.

[29] Panday B, Yamamoto A, Morita K (2003). Serodiagnosis of Japanese encephalitis among Nepalese patients by the particle agglutination assay. *Epidemiology and Infection*.

[30] Gadkari DA, Shaikh BH (1984). IgM antibody capture ELISA in the diagnosis of Japanese encephalitis, West Nile & dengue virus infections. *The Indian Journal of Medical Research*.

[31] Khalakdina A, Shrestha SK, Malla S (2010). Field evaluation of commercial Immunoglobulin M antibody capture ELISA diagnostic tests for the detection of Japanese encephalitis virus infection among encephalitis patients in Nepal. *International Journal of Infectious Diseases*.

[32] Anuradha SK, Surekha YA, Narayan S (2011). Japanese encephalitis virus: common cause of viral encephalitis in paediatric age group in Bellary, Karnataka, India. *Journal of Clinical and Diagnostic Research*.

[33] Koene MGJ, Mulder HA, Stockhofe-Zurwieden N, Kruijt L, Smits MA (2012). Serum protein profiles as potential biomarkers for infectious disease status in pigs. *BMC Veterinary Research*.

[34] Favoreel HW, van de Walle GR, Nauwynck HJ, Pensaert MB (2003). Virus component evasion strategies. *Journal of General Virology*.

[35] Kantake M (2011). Blood, urine and CSF analysis. *Nippon Rinsho*.

[36] Misra UK, Srivastava R, Kalita J, Khan MY (2010). Sequential changes in serum cytokines and chemokines in a rat model of Japanese encephalitis. *NeuroImmunomodulation*.

[37] Behrens G, Li M, Smith CM (2004). Helper T cells, dendritic cells and CTL immunity. *Immunology and Cell Biology*.

[38] Kopf M, Abel B, Gallimore A, Carroll M, Bachmann MF (2002). Complement component C3 promotes T-cell priming and lung migration to control acute influenza virus infection. *Nature Medicine*.

[39] Carroll MC (2004). The complement system in regulation of adaptive immunity. *Nature Immunology*.

[40] Lindahl G, Sjöbring U, Johnsson E (2000). Human complement regulators: a major target for pathogenic microorganisms. *Current Opinion in Immunology*.

[41] Muller-Eberhard HJ (1988). Molecular organization and function of the complement system. *Annual Review of Biochemistry*.

[42] Stein JV, Nombela-Arrieta C (2005). Chemokine control of lymphocyte trafficking: a general overview. *Immunology*.

[43] Guabiraba R, Marques RE, Besnard A-G (2010). Role of the chemokine receptors CCR1, CCR2 and CCR4 in the pathogenesis of experimental dengue infection in mice. *PLoS ONE*.

[44] Kliks SC, Nisalak A, Brandt WE, Wahl L, Burke DS (1989). Antibody-dependent enhancement of dengue virus growth in human monocytes as a risk factor for dengue hemorrhagic fever. *American Journal of Tropical Medicine and Hygiene*.

[45] Imhof BA, Aurrand-Lions M (2004). Adhesion mechanisms regulating the migration of monocytes. *Nature Reviews Immunology*.

[46] Atemezem A, Mbemba E, Vassy R, Slimani H, Saffar L, Gattegno L (2001). Human *α*1-acid glycoprotein binds to CCR5 expressed on the plasma membrane of human primary macrophages. *Biochemical Journal*.

[47] Bhatt GC, Bondre VP, Sapkal GN (2012). Changing clinico-laboratory profile of encephalitis patients in the eastern Uttar Pradesh region of India. *Tropical Doctor*.

[48] Kunkel EJ, Butcher EC (2002). Chemokines and the tissue-specific migration of lymphocytes. *Immunity*.

[49] Cyster JG (2005). Chemokines, sphingosine-1-phosphate, and cell migration in secondary lymphoid organs. *Annual Review of Immunology*.

[50] Gabaj C, Kushner I (1999). Acute-phase proteins and other systemic responses to inflammation. *The New England Journal of Medicine*.

[51] Steel DM, Whitehead AS (1994). The major acute phase reactants: C-reactive protein, serum amyloid P component and serum amyloid A protein. *Immunology Today*.

[52] Kim CH (2005). The greater chemotactic network for lymphocyte trafficking: chemokines and beyond. *Current Opinion in Hematology*.

[53] Reth M (1995). The B-cell antigen receptor complex and co-receptors. *Immunology Today*.

[54] Kurane I, Innis BL, Nisalak A (1989). Human T cell response to dengue virus antigens. Proliferative responses and interferon gamma production. *Journal of Clinical Investigation*.

[55] Kurane I, Innis BL, Hoke CH (1995). T cell activation in vivo by dengue virus infection. *Journal of Clinical & Laboratory Immunology*.

[56] Stanfield RL, Wilson IA (1995). Protein-peptide interactions. *Current Opinion in Structural Biology*.

[57] Mishra MK, Koli P, Bhowmick S, Basu A (2007). Neuroprotection conferred by astrocytes is insufficient to protect animals from succumbing to Japanese encephalitis. *Neurochemistry International*.

[58] Hase T, Summers PL, Dubois DR (1990). Ultrastructural changes of mouse brain neurons infected with Japanese encephalitis virus. *International Journal of Experimental Pathology*.

[59] Su H-L, Liao C-L, Lin Y-L (2002). Japanese encephalitis virus infection initiates endoplasmic reticulum stress and an unfolded protein response. *Journal of Virology*.

[60] Maschke M, Kastrup O, Forsting M, Diener H-C (2004). Update on neuroimaging in infectious central nervous system disease. *Current Opinion in Neurology*.

[61] Xie W, Schlücker S (2013). Medical applications of surface-enhanced Raman scattering. *Physical Chemistry Chemical Physics*.

[62] Nordberg A (2004). PET imaging of amyloid in Alzheimer’s disease. *The Lancet Neurology*.

[63] Myint KSA, Gibbons RV, Perng GC, Solomon T (2007). Unravelling the neuropathogenesis of Japanese encephalitis. *Transactions of the Royal Society of Tropical Medicine and Hygiene*.

[64] Hills SL, Phillips DC (2009). Past, present, and future of Japanese encephalitis. *Emerging Infectious Disease Journal*.

[65] Feuer R, Mena I, Pagarigan RR, Harkins S, Hassett DE, Whitton JL (2003). Coxsackievirus B3 and the neonatal CNS: the roles of stem cells, developing neurons, and apoptosis in infection, viral dissemination, and disease. *American Journal of Pathology*.

[66] Feuer R, Pagarigan RR, Harkins S, Liu F, Hunziker IP, Whitton JL (2005). Coxsackievirus targets proliferating neuronal progenitor cells in the neonatal CNS. *Journal of Neuroscience*.

[67] Kosugi I, Shinmura Y, Kawasaki H (2000). Cytomegalovirus infection of the central nervous system stem cells from mouse embryo: a model for developmental brain disorders induced by cytomegalovirus. *Laboratory Investigation*.

[68] Das S, Basu A (2008). Inflammation: a new candidate in modulating adult neurogenesis. *Journal of Neuroscience Research*.

[69] Das S, Basu A (2008). Japanese encephalitis virus infects neural progenitor cells and decreases their proliferation. *Journal of Neurochemistry*.

[70] Ghoshal A, Das S, Ghosh S (2007). Proinflammatory mediators released by activated microglia induces neuronal death in Japanese encephalitis. *GLIA*.

[71] Swarup V, Das S, Ghosh S, Basu A (2007). Tumor necrosis factor receptor-1-induced neuronal death by TRADD contributes to the pathogenesis of Japanese encephalitis. *Journal of Neurochemistry*.

[72] Wang JJ, Liao CL, Yang CI, Lin YL, Chiou CT, Chen LK (1998). Localizations of NS3 and E proteins in mouse brain infected with mutant strain of Japanese encephalitis virus. *Archives of Virology*.

[73] Yang T-C, Shiu S-L, Chuang P-H (2009). Japanese encephalitis virus NS2B-NS3 protease induces caspase 3 activation and mitochondria-mediated apoptosis in human medulloblastoma cells. *Virus Research*.

[74] Mishra MK, Basu A (2008). Minocycline neuroprotects, reduces microglial activation, inhibits caspase 3 induction, and viral replication following Japanese encephalitis. *Journal of Neurochemistry*.

[75] Deng X, Shi Z, Li S (2011). Characterization of nonstructural protein 3 of a neurovirulent Japanese encephalitis virus strain isolated from a pig. *Virology Journal*.

[76] Vrati S, Agarwal V, Malik P, Wani SA, Saini M (1999). Molecular characterization of an Indian isolate of Japanese encephalitis virus that shows an extended lag phase during growth. *Journal of General Virology*.

[77] Yasui K (2002). Neuropathogenesis of Japanese encephalitis virus. *Journal of NeuroVirology*.

[78] Reynolds BA, Weiss S (1996). Clonal and population analyses demonstrate that an EGF-responsive mammalian embryonic CNS precursor is a stem cell. *Developmental Biology*.

[79] Felling RJ, Snyder MJ, Romanko MJ (2006). Neural stem/progenitor cells participate in the regenerative response to perinatal hypoxia/ischemia. *Journal of Neuroscience*.

[80] Schmid I, Cole SW, Korin YD, Zack JA, Giorgi JV (2000). Detection of cell cycle subcompartments by flow cytometric estimation of DNA-RNA content in combination with dual-color immunofluorescence. *Cytometry*.

[81] Morrison SJ, Shah NM, Anderson DJ (1997). Regulatory mechanisms in stem cell biology. *Cell*.

[82] Sooryanarain H, Ayachit V, Gore M (2012). Activated CD56(+) lymphocytes (NK+NKT) mediate immunomodulatory and anti-viral effects during Japanese encephalitis virus infection of dendritic cells in vitro. *Virology*.

[83] Aleyas AG, Han YW, George JA (2010). Multifront assault on antigen presentation by Japanese encephalitis virus subverts CD8+ T cell responses. *Journal of Immunology*.

[84] Aleyas AG, Han YW, Patil AM (2012). Impaired cross-presentation of CD8*α*+ CD11c+ dendritic cells by Japanese encephalitis virus in a TLR2/MyD88 signal pathway dependent manner. *European Journal of Immunology*.

[85] Zhai YZ, Zhou Y, Ma L, Feng GH (2013). The dominant roles of ICAM-1-endcoding gene in DNA vaccination against Japanese encephalitis virus are the activation of dendritic cells and enhancement of cellular immunity. *Cellular Immunology*.

[86] Thongtan T, Wikan N, Wintachai P (2012). Characterization of putative Japanese encephalitis virus receptor molecules on microglial cells. *Journal of Medical Virology*.

[87] Chen C-J, Ou Y-C, Chang C-Y (2011). TNF-*α* and IL-1*β* mediate Japanese encephalitis virus-induced RANTES gene expression in astrocytes. *Neurochemistry International*.

[88] Mishra MK, Koli P, Bhowmick S, Basu A (2007). Neuroprotection conferred by astrocytes is insufficient to protect animals from succumbing to Japanese encephalitis. *Neurochemistry International*.

[89] van de Weg CA, de Araújo Es, van den Ham HJ (2013). Microbial translocation is associated with extensive immune activation in dengue virus infected patients with severe disease. *PLOS Neglected Tropical Diseases*.

[90] Gupta N, Lomash V, Rao PVL (2010). Expression profile of Japanese encephalitis virus induced neuroinflammation and its implication in disease severity. *Journal of Clinical Virology*.

[91] Tseng YF, Wang CC, Liao SK, Chuang CK, Chen WJ (2011). Automimmunity related demyelination in infection by Japanese encephalitis virus. *Journal of Biomedical Science*.

[92] Mishra MK, Dutta K, Saheb SK, Basu A (2009). Understanding the molecular mechanism of blood-brain barrier damage in an experimental model of Japanese encephalitis: correlation with minocycline administration as a therapeutic agent. *Neurochemistry International*.

[93] Tanuma N (2012). CSF biomarekrs in children with acute encephalopathy syndrome. *Nihon Rinsho*.

[94] Argade SP, Banerjee K (1990). Plasma lactic dehydrogenase in mice infected with Japanese encephalitis and West Nile viruses. *Indian Journal of Medical Research A*.

[95] Lai YC qui CY, Chang CY, Pan HC (2012). Endothelial Japanese encephalitis virus infection enhances migration and adhesion of leukocytes to brain microvascular endothelia via MEK-dependent expression of ICAM1 and the CINC and RANTES chemokines. *Journal of Neurochemistry*.

[96] Bardina SV, Lim JK (2012). The role of chemokines in the pathogenesis of neurotropic flaviviruses. *Immunologic Research*.

[97] Yang T-C, Lai C-C, Shiu S-L (2010). Japanese encephalitis virus down-regulates thioredoxin and induces ROS-mediated ASK1-ERK/p38 MAPK activation in human promonocyte cells. *Microbes and Infection*.

[98] Valyi-Nagy T, Dermody TS (2005). Role of oxidative damage in the pathogenesis of viral infections of the nervous system. *Histology and Histopathology*.

[99] Srivastava R, Kalita J, Khan MY, Misra UK (2009). Free radical generation by neurons in rat model of Japanese encephalitis. *Neurochemical Research*.

[100] Cao S, Li Y, Ye J (2011). Japanese encephalitis virus wild strain infection suppresses dendritic cells maturation and function, and causes the expansion of regulatory T cells. *Virology Journal*.

[101] Sinniah M (1989). A review of Japanese-B virus encephalitis in Malaysia. *The Southeast Asian Journal of Tropical Medicine and Public Health*.

[102] Kang B, Oh J, Lee C (2007). Evaluation of a rapid immunodiagnostic test kit for rabies virus. *Journal of Virological Methods*.

[103] Gould EA, Buckley A (1989). Antibody-dependent enhancement of yellow fever and Japanese encephalitis virus neurovirulence. *Journal of General Virology*.

[104] Gould EA, Buckley A, Groeger BK, Cane PA, Doenhoff M (1987). Immune enhancement of yellow fever virus neurovirulence for mice: Studies of mechanisms involved. *Journal of General Virology*.

[105] Yang KD, Yeh W-T, Chen R-F (2004). A model to study neurotropism and persistency of Japanese encephalitis virus infection in human neuroblastoma cells and leukocytes. *Journal of General Virology*.

[106] Kumar Y, Liang, Bo Z, Rajapakse JC, Ooi EE, Tannenbaum SR (2012). Serum proteome and cytokine analysis in a longitudinal cohort of adults with primary dengue infection reveals predictive markers of DHF. *PLOS Neglected Tropical Diseases*.

[107] Endy TP, Nisalak A (2002). Japanese encephalitis virus: ecology and epidemiology. *Current Topics in Microbiology and Immunology*.

[108] Gromeier M, Wimmer E, Gorbalenya AE, Domingo E, Webster RG, Holland JJ (1999). Genetics, pathogenesis and evolution of picornaviruses. *Origin and Evolution of Viruses*.

[109] Chambers TJ, Hahn CS, Galler R, Rice CM (1990). Flavivirus genome organization, expression, and replication. *Annual Review of Microbiology*.

[110] Lin C, Amberg SM, Chambers TJ, Rice CM (1993). Cleavage at a novel site in the NS4A region by the yellow fever virus NS2B-3 proteinase is a prerequisite for processing at the downstream 4A/4B signalase site. *Journal of Virology*.

[111] Rice CM, Lenches EM, Eddy SR (1985). Nucleotide sequence of yellow fever virus: implications for flavivirus gene expression and evolution. *Science*.

[112] Rice CM, Strauss EG, Strauss JH, Schlesinger S, Schlesinger MJ (1986). Structure of the flavivirus genome. *The Togaviridae and Flaviviridae*.

[113] Sumiyoshi H, Mori C, Fuke I (1987). Complete nucleotide sequence of the Japanese encephalitis virus genome RNA. *Virology*.

[114] Yamashita T, Unno H, Mori Y (2008). Crystal structure of the catalytic domain of Japanese encephalitis virus NS3 helicase/nucleoside triphosphatase at a resolution of 1.8 Å. *Virology*.

[115] Muller DA, Young PR (2013). The flavivirus NS1 protein: molecular and structural biology, immunology, role in pathogenesis and application as a diagnostic biomarker. *Antiviral Research*.

[116] Deng Y-Q, Dai J-X, Ji G-H (2011). A broadly flavivirus cross-neutralizing monoclonal antibody that recognizes a novel epitope within the fusion loop of E protein. *PLoS ONE*.

[117] Heinz FX, Berger R, Tuma W, Kunz C (1983). A topological and functional model of epitopes on the structural glycoprotein of tick-borne encephalitis virus defined by monoclonal antibodies. *Virology*.

[118] Gould EA, Buckley A, Barrett ADT, Cammack N (1986). Neutralizing (54K) and non-neutralizing (54K and 48K) monoclonal antibodies against structural and non-structural yellow fever virus proteins confer immunity in mice. *Journal of General Virology*.

[119] Brandriss MW, Schlesinger JJ, Walsh EE, Briselli M (1986). Lethal 17D yellow fever encephalitis in mice. I. Passive protection by monoclonal antibodies to the envelope proteins of 17D yellow fever and dengue 2 viruses. *Journal of General Virology*.

[120] Muller DA, Corrie SR, Coffey J, Young PR, Kendall MA (2012). Surface modified microprojection arrays for the selective extraction of the dengue virus NS1 protein as a marker for disease. *Analytical Chemistry*.

[121] Muller DA, Frentiu FD, Rojas A, Moreira LA, O’Neill SL, Young PR (2012). A portable approach for the surveillance of dengue virus-infected mosquitoes. *Journal of Virological Methods*.

[122] Schlesinger JJ, Brandriss MW, Walsh EE (1987). Protection of mice against dengue 2 virus encephalitis by immunization with the dengue 2 virus non-structural glycoprotein NS1. *Journal of General Virology*.

[123] Henchal EA, Henchal LS, Thaisomboonsuk BK (1987). Topological mapping of unique epitopes on the dengue-2 virus NS1 protein using monoclonal antibodies. *Journal of General Virology*.

[124] Falgout B, Bray M, Schlesinger JJ, Lai C-J (1990). Immunization of mice with recombinant vaccinia virus expressing authentic dengue virus nonstructural protein NS1 protects against lethal dengue virus encephalitis. *Journal of Virology*.

[125] Avirutnan P, Punyadee N, Noisakran S (2006). Vascular leakage in severe dengue virus infections: a potential role for the nonstructural viral protein NS1 and complement. *Journal of Infectious Diseases*.

[126] Sun D-S, King C-C, Huang H-S (2007). Antiplatelet autoantibodies elicited by dengue virus non-structural protein 1 cause thrombocytopenia and mortality in mice. *Journal of Thrombosis and Haemostasis*.

[127] Bray M, Zhao B, Markoff L, Eckels KH, Chanock RM, Lai C-J (1989). Mice immunized with recombinant vaccinia virus expressing dengue 4 virus structural proteins with or without nonstructural protein NS1 are protected against fatal dengue virus encephalitis. *Journal of Virology*.

[128] Lin Y-L, Chen L-K, Liao C-L (1998). Protective immunity in mice virus nonstructural protein NS1 elicits DNA immunization with Japanese encephalitis. *Journal of Virology*.

[129] Konishi E, Pincus S, Fonseca BAL, Shope RE, Paoletti E, Mason PW (1991). Comparison of protective immunity elicited by recombinant vaccinia viruses that synthesize E or NS1 of Japanese encephalitis virus. *Virology*.

[130] Schlesinger JJ, Brandriss MW, Walsh EE (1985). Protection against 17D yellow fever encephalitis in mice by passive transfer of monoclonal antibodies to the nonstructural glycoprotein gp48 and by active immunization with gp48. *Journal of Immunology*.

[131] Schlesinger JJ, Brandriss MW, Cropp CB, Monath TP (1986). Protection against yellow fever in monkeys by immunization with yellow fever virus nonstructural protein NS1. *Journal of Virology*.

[132] Kawano H, Rostapshov V, Rosen L, Lai C-J (1993). Genetic determinants of dengue type 4 virus neurovirulence for mice. *Journal of Virology*.

[133] Mackenzie JM, Jones MK, Young PR (1996). Immunolocalization of the Dengue virus nonstructural glycoprotein NS1 suggests a role in viral RNA replication. *Virology*.

[134] Westaway EG, Mackenzie JM, Kenney MT, Jones MK, Khromykh AA (1997). Ultrastructure of Kunjin virus-infected cells: colocalization of NS1 and NS3 with double-stranded RNA, and of NS2B with NS3, in virus-induced membrane structures. *Journal of Virology*.

[135] Winkler G, Randolph VB, Cleaves GR, Ryan TE, Stollar V (1988). Evidence that the mature form of the flavivirus nonstructural protein NS1 is a dimer. *Virology*.

[136] Mason PW (1989). Maturation of Japanese encephalitis virus glycoproteins produced by infected mammalian and mosquito cells. *Virology*.

[137] Smith GW, Wright PJ (1985). Synthesis of proteins and glycoproteins in dengue type 2 virus-infected vero and *Aedes albopictus* cells. *Journal of General Virology*.

[138] Westaway EG, Goodman MR (1987). Variation in distribution of the three flavivirus-specified glycoproteins detected by immunofluorescence in infected vero cells. *Archives of Virology*.

[139] Gutsche I, Coulibaly F, Voss JE (2011). Secreted dengue virus nonstructural protein NS1 is an atypical barrel-shaped high-density lipoprotein. *Proceedings of the National Academy of Sciences of the United States of America*.

[140] Chen L-K, Liao C-L, Lin C-G (1996). Persistence of Japanese encephalitis virus is associated with abnormal expression of the nonstructural protein NS1 in host cells. *Virology*.

[141] Hung J-J, Hsieh M-T, Young M-J, Kao C-L, King C-C, Chang W (2004). An external loop region of domain III of dengue virus type 2 envelope protein is involved in serotype-specific binding to mosquito but not mammalian cells. *Journal of Virology*.

[142] Goto A, Yoshii K, Obara M (2005). Role of the N-linked glycans of the prM and E envelope proteins in tick-borne encephalitis virus particle secretion. *Vaccine*.

[143] Wei H-Y, Jiang L-F, Fang D-Y, Guo H-Y (2003). Dengue virus type 2 infects human endothelial cells through binding of the viral envelope glycoprotein to cell surface polypeptides. *Journal of General Virology*.

[144] Jeong MK, Sang IY, Byung HS A single-linked glycosilation site in the Japanese encephalitis virus prM protein is critical for cell type-specific prM protein biogenesis, virus particle release and pathogenicity in mice. *Journal of Virology*.

[145] Gromeier M, Bossert B, Arita M, Nomoto A, Wimmer E (1999). Dual stem loops within the poliovirus internal ribosomal entry site control neurovirulence. *Journal of Virology*.

[146] Zhang S-B, Li P, Liu X-Z (2010). Analysis on neutralization antibody titer of Japanese B encephalitis virus in healthy population in Shaanxi province. *Zhongguo Yi, Miao He Mian Yi*.

[147] Bray M, Lai C-J (1991). Construction of intertypic chimeric dengue viruses by substitution of structural protein genes. *Proceedings of the National Academy of Sciences of the United States of America*.

[148] Johnston LJ, Halliday GM, King NJ (2000). Langerhans cells are targets of dengue virus infection. *Nature Medicine*.

[149] Zhang Y, Chen P, Cao R, Gu J (2011). Mutation of putative N-linked glycosylation sites in japanese encephalitis virus premembrane and envelope proteins enhances humoral immunity in BALB/C mice after DNA vaccination. *Virology Journal*.

[150] Zhang M, Gaschen B, Blay W (2004). Tracking global patterns of N-linked glycosylation site variation in highly variable viral glycoproteins: HIV, SIV, and HCV envelopes and influenza hemagglutinin. *Glycobiology*.

[151] Goffard A, Callens N, Bartosch B (2005). Role of N-linked glycans in the functions of hepatitis C virus envelope glycoproteins. *Journal of Virology*.

[152] Schlesinger RW, Schlesinger RW (1980). Virus-host interactions in natural and experimental infections with alphaviruses and flaviviruses. *The Togaviruses*.

[153] Agol VI, Drozdov SG, Grachev VP (1985). Recombinants between attenuated and virulent strains of poliovirus type 1: derivation and characterization of recombinants with centrally located crossover points. *Virology*.

[154] Omata T, Kohara M, Kuge S (1986). Genetic analysis of the attenuation phenotype of poliovirus type 1. *Journal of Virology*.

[155] Tardy-Panit M, Blondel B, Martin A, Tekaia F, Horaud F, Delpeyroux F (1993). A mutation in the RNA polymerase of poliovirus type 1 contributes to attenuation in mice. *Journal of Virology*.

[156] Thomas P, Monath JA, Juan A (2002). Single mutation in the flavivirus envelope protein hinge region increases neurovirulence for mice and monkeys but decreases viscerotropism for monkeys: relevance to development and safety testing of live, attenuated vaccines. *Journal of Virology*.

[157] Ahmed M, Marino TR, Puckett S, Kock ND, Lyles DS (2008). Immune response in the absence of neurovirulence in mice infected with M protein mutant vesicular stomatitis virus. *Journal of Virology*.

[158] Schlesinger JJ, Chapman S, Nestorowicz A, Rice CM, Ginocchio TE, Chambers TJ (1996). Replication of yellow fever virus in the mouse central nervous system: comparison of neuroadapted and non-neuroadapted virus and partial sequence analysis of the neuroadapted strain. *Journal of General Virology*.

[159] Feuer R, Mena I, Pagarigan RR, Harkins S, Hassett DE, Whitton JL (2003). Coxsackievirus B3 and the neonatal CNS: the roles of stem cells, developing neurons, and apoptosis in infection, viral dissemination, and disease. *American Journal of Pathology*.

[160] Feuer R, Pagarigan RR, Harkins S, Liu F, Hunziker IP, Whitton JL (2005). Coxsackievirus targets proliferating neuronal progenitor cells in the neonatal CNS. *Journal of Neuroscience*.

[161] Yang Y, Ye J, Yang X, Jiang R, Chen H, Cao S (2011). Japanese encephalitis virus infection induces changes of mRNA profile of mouse spleen and brain. *Virology Journal*.

[162] Sahoo GC, Dikhit MR, Das P (2008). Functional assignment to JEV proteins using SVM. *Bioinformation*.

[163] Saini M, Vrati S (2003). A Japanese encephalitis virus peptide present on Johnson grass mosaic virus-like particles induces virus-neutralizing antibodies and protects mice against lethal challenge. *Journal of Virology*.

[164] Konishi E, Yamaoka M, Kurane I, Mason PW (2000). Japanese encephalitis DNA vaccine candidates expressing premembrane and envelope genes induce virus-specific memory B cells and long-lasting antibodies in swine. *Virology*.

[165] Wu S-C, Lin C-W (2001). Neutralizing peptide ligands selected from phage-displayed libraries mimic the conformational epitope on domain III of the Japanese encephalitis virus envelope protein. *Virus Research*.

[166] Liu M, Chen H, Luo F (2007). Deletion of N-glycosylation sites of hepatitis C virus envelope protein E1 enhances specific cellular and humoral immune responses. *Vaccine*.

[167] Lin C-W, Cheng C-W, Yang T-C (2008). Interferon antagonist function of Japanese encephalitis virus NS4A and its interaction with DEAD-box RNA helicase DDX42. *Virus Research*.

[168] Monath TP, Arroyo J, Levenbook I (2002). Single mutation in the flavivirus envelope protein hinge region increases neurovirulence for mice and monkeys but decreases viscerotropism for monkeys: relevance to development and safety testing of live, attenuated vaccines. *Journal of Virology*.

[169] Arroyo J, Guirakhoo F, Fenner S, Zhang Z-X, Monath TP, Chambers TJ (2001). Molecular basis for attenuation of neurovirulence of a yellow fever virus/Japanese encephalitis virus chimera vaccine (ChimeriVax-JE). *Journal of Virology*.

[170] Yang D-K, Kweon C-H, Kim B-H (2004). Taqman reverse transcription polymerase chain reaction for the detection of Japanese encephalitis virus. *Journal of Veterinary Science*.

[171] Paranjpe S, Walimbe A, Banerjee K (1997). Statistical analysis of the envelope gene and the prM region of Japanese encephalitis virus: evidence suggestive of positive selection. *Journal of Genetics*.

[172] Appaiahgari MB, Vrati S (2007). DNAzyme-mediated inhibition of Japanese encephalitis virus replication in mouse brain. *Molecular Therapy*.

[173] Yang Y, Ye J, Yang X, Jiang R, Chen H, Cao S (2011). Japanese encephalitis virus infection induces changes of mRNA profile of mouse spleen and brain. *Virol J*.

[174] Zhang Y, Jia Y, Zheng R (2010). Plasma microRNA-122 as a biomarker for viral-, alcohol-, and chemical-related hepatic diseases. *Clinical Chemistry*.

[175] Skalsky RL, Cullen BR (2010). Viruses, microRNAs, and host interactions. *Annual Review of Microbiology*.

[176] Turchinovich A, Weiz L, Langheinz A, Burwinkel B (2011). Characterization of extracellular circulating microRNA. *Nucleic Acids Research*.

[177] Gupta A, Nagilla P, Le H-S (2011). Comparative expression profile of miRNA and mRNA In primary peripheral blood mononuclear cells infected with human immunodeficiency virus (HIV-1). *PLoS ONE*.

[178] Prescott MA, Pastey MK (2010). Identification of unique blood and urine biomarkers in influenza virus and *Staphylococcus aureus* co-infection: a preliminary study. *Biomarker Insights*.

[179] Swarup V, Ghosh J, Mishra MK, Basu A (2008). Novel strategy for treatment of Japanese encephalitis using arctigenin, a plant lignan. *Journal of Antimicrobial Chemotherapy*.

[180] Dutta K, Ghosh D, Basu A (2009). Curcumin protects neuronal cells from japanese encephalitis virus-mediated cell death and also inhibits infective viral particle formation by dysregulation of ubiquitin-proteasome system. *Journal of Neuroimmune Pharmacology*.

[181] O E, Era, Askling HH (2012). Cross protective capacity of Japanese encephalitis (JE) vaccine against circulating heterologous JE virus genotypes. *Clinical Infectious Diseases*.

[182] Holmskov U, Thiel S, Jensenius JC (2003). Collectins and ficolins: humoral lectins of the innate immune defense. *Annual Review of Immunology*.

[183] Rosen SD (2004). Ligands for L-selectin: homing, inflammation, and beyond. *Annual Review of Immunology*.

[184] Turner MW (2003). The role of mannose-binding lectin in health and disease. *Molecular Immunology*.

[185] Yamada K, Mano T, Inada Y (2011). Changes of brain edema after initiation of mild hypothermia therapy in children. *No To Hattatsu*.

[186] Zhang J-S, Zhao Q-M, Zuo S-Q, Jia N, Guo X-F (2012). Cytokine and chemokine responses to Japanese encephalitis live attenuated vaccine in a human population. *International Journal of Infectious Diseases*.

[187] Takegami T, Miyamoto H, Nakamura H, Yasui K (1982). Biological activities of the structural proteins of Japanese encephalitis virus. *Acta Virologica*.

[188] Appaiahgari MB, Vrati S (2007). DNAzyme-mediated inhibition of Japanese encephalitis virus replication in mouse brain. *Molecular Therapy*.

[189] Yun S-I, Kim S-Y, Rice CM, Lee Y-M (2003). Development and application of a reverse genetics system for Japanese encephalitis virus. *Journal of Virology*.

[190] Lin Y-L, Huang Y-L, Ma S-H (1997). Inhibition of Japanese encephalitis virus infection by nitric oxide: antiviral effect of nitric oxide on RNA virus replication. *Journal of Virology*.

[191] Kumar P, Sang KL, Shankar P, Manjunath N (2006). A single siRNA suppresses fatal encephalitis induced by two different flaviviruses. *PLoS Medicine*.

[192] Upadhyay RK (2013). Japanese encephalitis virus generated neurovirulence, antigenicity, and host immune responses. *ISRN Virology*.

[193] Upadhyay RK, Ahmad S (2011). Japanese encephalitis virus: its epidemiology, disease and vector control with special reference to immune surveillance and safety measures. *Journal of Pharmacy Research*.

[194] Kumari R, Kumar K, Rawat G A, Singh, Yadav NK, Chauhan LS (2013). First indigenous transmission of Japanese encephalitis in urban areas of National Capital Territory of Delhi. India. *Tropical Medicine & International Health*.

